# Data-collection strategy for challenging native SAD phasing

**DOI:** 10.1107/S2059798315024110

**Published:** 2016-03-01

**Authors:** Vincent Olieric, Tobias Weinert, Aaron D. Finke, Carolin Anders, Dianfan Li, Natacha Olieric, Camelia N. Borca, Michel O. Steinmetz, Martin Caffrey, Martin Jinek, Meitian Wang

**Affiliations:** a Swiss Light Source, Paul Scherrer Institut, Villigen PSI, Switzerland; b Department of Biochemistry, University of Zurich, Zurich, Switzerland; c Membrane Structural and Functional Group, Schools of Medicine and Biochemistry and Immunology, Trinity College, Dublin, Ireland; d Laboratory of Biomolecular Research, Department of Biology and Chemistry, Paul Scherrer Institut, Villigen PSI, Switzerland

**Keywords:** native SAD phasing, anomalous signal, data-collection strategy, complex, membrane protein

## Abstract

Recent improvements in data-collection strategies have pushed the limits of native SAD (single-wavelength anomalous diffraction) phasing, a method that uses the weak anomalous signal of light elements naturally present in macromolecules. These involve the merging of multiple data sets from either multiple crystals or from a single crystal collected in multiple orientations at a low X-ray dose. Both approaches yield data of high multiplicity while minimizing radiation damage and systematic error, thus ensuring accurate measurements of the anomalous differences. Here, the combined use of these two strategies is described to solve cases of native SAD phasing that were particular challenges: the integral membrane diacylglycerol kinase (DgkA) with a low Bijvoet ratio of 1% and the large 200 kDa complex of the CRISPR-associated endonuclease (Cas9) bound to guide RNA and target DNA crystallized in the low-symmetry space group *C*2. The optimal native SAD data-collection strategy based on systematic measurements performed on the 266 kDa multiprotein/multiligand tubulin complex is discussed.

## Abstract 2

The successful structure solution of the integral membrane diacylglycerol kinase and the CRISPR-associated endonuclease RNA–DNA complex by native SAD phasing is demonstrated. The structures were solved with a combined low-dose multi-orientation, multi-crystal data-collection strategy.

## 1. Introduction   

Native SAD (single-wavelength anomalous diffraction) is a *de novo* macromolecular structure-determination method in which the phase problem is solved by exploiting the anomalous diffraction signal of light atoms present in the crystal (for a review of anomalous diffraction, see Hendrickson, 2014 ▸). Unlike other *de novo* methods, structure solution by native SAD does not require the incorporation of exogenous heavy atoms, which can be tedious and can lead to non-isomorphism. However, native SAD phasing has its own challenges. Firstly, the absorption edge, where anomalous scattering is maximized, is below 2.5 keV (>5 Å) for elements such as sulfur and phosphorus, which is beyond the reach of most current macromolecular crystallography (MX) synchrotron beamlines. Data collection is also hindered by sample and air absorption of X-rays at such low energies. Thus, native SAD data collection is usually performed at ‘compromise’ energies of around 6 keV (2.066 Å; Mueller-Dieckmann *et al.*, 2005 ▸; Liu *et al.*, 2014 ▸; Weinert *et al.*, 2015 ▸) to maximize the anomalous signal while minimizing absorption. Alternatively, home sources, which typically operate with Cu *K*α (1.54 Å) or Cr *K*α (2.29 Å) radiation, can also be used for such measurements. Secondly, because data collection is performed far from the absorption edges of these light elements, the resulting anomalous signal is small and accurate measurements of the reflection intensities and their differences are essential. To obtain this level of accuracy, random and systematic errors must be minimized. However, typical data-collection protocols at third-generation synchrotrons, usually performed around a single axis, yield anomalous data that can be adversely affected by radiation damage if insufficient care is given to the X-ray dose delivered to the crystal. Consequently, successful cases of phasing by native SAD remain rare; currently, about 150 native SAD structures have been deposited in the Protein Data Bank (Berman *et al.*, 2000 ▸).

Recent advances in data-collection strategies have breathed new life into native SAD phasing (reviewed by Rose *et al.*, 2015 ▸). The multi-crystal averaging approach involves merging data sets from statistically equivalent crystals (Liu *et al.*, 2012 ▸, 2013 ▸), enhancing the anomalous signal-to-noise ratio while minimizing anomalous signal decay owing to radiation damage. The key to successful multi-crystal averaging having enough isomorphous crystals available for data collection. This method is possible because data-merging and data-processing algorithms are robust enough to accommodate a large number of distinct crystal data sets. In particularly challenging cases, data sets from as many as 18 (Akey *et al.*, 2014 ▸) and 32 (El Omari *et al.*, 2014 ▸) separate crystals were required to obtain sufficient anomalous signal for successful phasing. An extreme case is the recent native SAD phasing of lysozyme using serial crystallography data collected at room temperature, where 2° of data obtained individually from 992 randomly oriented crystals with an average size of 20 µm were merged (Huang *et al.*, 2015 ▸). A second approach involves collecting many low-dose data sets (<0.5 MGy per 360°) from a single crystal in multiple orientations, mitigating radiation damage while also reducing systematic errors in data collection (Debreczeni *et al.*, 2003 ▸; Brockhauser *et al.*, 2013 ▸; Weinert *et al.*, 2015 ▸; Finke *et al.*, 2016 ▸). By varying the crystal orientation, it is possible to measure the same reflections in different diffraction geometries on different areas of the detector and with different sample absorption, thereby reducing systematic error. This approach has proven to be successful for 11 real-life cases, including a 266 kDa multiprotein/multiligand complex, the largest structure solved by native SAD to date (Weinert *et al.*, 2015 ▸). The method benefited from instrumentation developments such as a high-precision PRIGo multi-axis goniometer (Waltersperger *et al.*, 2015 ▸) and a photon-counting, noise-free pixel-array detector calibrated for low energies (Henrich *et al.*, 2009 ▸), both of which were used in the current study.

Despite the many recent improvements in data-collection strategies, difficult cases such as large anomalous scattering substructures, low-symmetry space groups, low diffraction resolution, small crystals, low Bijvoet ratios or some combination thereof still remain a challenge for native SAD phasing. Fortunately, it is possible to combine the two strategies introduced above to extract as much anomalous signal as possible with the highest possible accuracy from as many crystals as needed for successful phasing (Klinke *et al.*, 2015 ▸). Here, we present two challenging native SAD cases solved by collecting low-dose data at multiple orientations from three crystals each: the integral membrane diacylglycerol kinase DgkA (Li *et al.*, 2013 ▸; 2 × 42 kDa asymmetric unit, space group *P*2_1_2_1_2_1_, 2.6 Å resolution) and the large Cas9–RNA–DNA complex (Anders *et al.*, 2014 ▸; 200 kDa asymmetric unit, space group *C*2, 2.2 Å resolution). In addition, we demonstrate the benefits of these combined low-dose multi-orientation, multi-crystal data-collection strategies using systematic measurements performed on the multiprotein/multiligand tubulin–RB3–tubulin tyrosine ligase complex T_2_R-TTL (Prota, Bargsten *et al.*, 2013 ▸; Weinert *et al.*, 2015 ▸; 266 kDa asymmetric unit, space group *P*2_1_2_1_2_1_, 2.3 Å resolution).

## 2. Materials and methods   

### 2.1. Crystallographic data collection, data processing and analysis   

All experiments were performed on the super-bending-magnet beamline X06DA (PXIII) at the Swiss Light Source (SLS), Villigen PSI, Switzerland operating at 2.4 GeV with 400 mA top-up mode. The beamline has a double channel-cut Si(111) monochromator with an energy resolution of 1.4 × 10^−4^ and an elliptical beam of 90 × 50 µm (FWHM). Data were collected at 100 K with a wavelength of 2.066 Å (6 keV) and a flux of ∼1.5 × 10^10^ photons s^−1^. Multi-orientation data collection was carried out at various χ and φ settings of the multi-axis PRIGo goniometer (Waltersperger *et al.*, 2015 ▸) and on a PILATUS 2M-F detector (Henrich *et al.*, 2009 ▸) operated in shutterless mode at a frame rate of 10 or 20 Hz and at a sample-to-detector distance of 120 mm. The data were processed using *XDS* and scaled and merged with *XSCALE* (Kabsch, 2010 ▸). The high-resolution data cutoff was based on the statistical indicators CC_1/2_ and CC* (Karplus & Diederichs, 2012 ▸). Substructure determination and phasing were performed with *SHELXC*/*D*/*E* (Sheldrick, 2010 ▸) using the *HKL*2*MAP* interface (Pape & Schneider, 2004 ▸). The anomalous peak heights were calculated using *AnoDe* without a resolution cutoff (Thorn & Sheldrick, 2011 ▸). The number of correct sites found with *SHELXD* was assessed using *SITCOM* (Dall’Antonia & Schneider, 2006 ▸). Refinement and model map cross-correlation calculations were performed using *PHENIX* (Adams *et al.*, 2010 ▸). X-ray dose was estimated with *RADDOSE*-3*D* (Zeldin *et al.*, 2013 ▸). The Bijvoet ratio was estimated using the formula
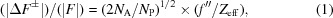
where *f*′′ is the imaginary scattering contribution of sulfur (0.95 e at 6 keV), *N*_A_ is the number of anomalous scattering atoms, *N*_P_ is the number of non-H atoms in the asymmetric unit, and *Z*_eff_ is the effective number of electrons of the average protein atom (6.7; Hendrickson & Teeter, 1981 ▸). Structure figures were prepared and rendered using *PyMOL* (DeLano, 2002 ▸).

#### 2.1.1. DgkA   

*P*2_1_2_1_2_1_ crystals of a DgkA mutant (A41C, C46A, I53V, I70L, M96L, V107D, C113A) measuring ∼100–200 × 50 × 20 µm (Supplementary Fig. S1*a*) were grown by the *in meso* or lipid cubic phase method as described elsewhere (Li *et al.*, 2013 ▸), harvested and snap-cooled in liquid nitrogen. Three crystals were used for data collection (Supplementary Fig. S1*a*). From the first crystal, which diffracted to 2.6 Å resolution, a total of ten 360° ω scans were collected with no change in orientation. The second crystal diffracted to 2.8 Å resolution and a total of eight 360° ω scans were collected, including two 360° ω scans with the crystal aligned along the a* axis in order to collect Bijvoet pairs simultaneously on the same image. Data for the third crystal, which diffracted to 2.8 Å resolution, were collected in multiple orientations: four 360° ω scans at χ = 0° and two 360° ω scans each at χ = 10, 20 and 30° (Table 1 ▸). At a data-collection speed of 1 deg s^−1^, the average dose per 360° ω scan was estimated to be 0.5 MGy. Coordinates and structure factors of DgkA have been deposited in the Protein Data Bank (PDB) under accession code 5dwk.

#### 2.1.2. Cas9–RNA–DNA   

Crystals of a Cas9–sgRNA–target DNA complex in which the complementary (target) DNA strand was mismatched to the sgRNA guide at positions 1–3 were generated, harvested and cryoprotected as described previously (Anders *et al.*, 2014 ▸). Three *C*2 crystals measuring ∼200 × 100 × 50 µm were used for data collection (Supplementary Fig. S1*b*). The first, second and third crystals diffracted to 2.2, 2.4 and 2.2 Å resolution, respectively, with anomalous signal extending to ∼2.9 Å resolution. The first crystal was large enough to collect two series of multi-orientation data sets from two well separated locations on the crystal (Supplementary Fig. S1*b*, crystal 1). At each location, a total of eight 360° ω scans were collected with varying χ/φ settings. For the second crystal, a total of eight 360° ω scans were collected at 5° χ increments from 0 to 35° while keeping the φ orientation constant. For the third crystal, four 360° ω scans were collected at φ = 0° and χ = 0, 10, 20 and 30°, and four 360° ω scans were collected at φ = 180° and χ = 0, 10, 20 and 30° (Table 1 ▸). At a data-collection speed of 2 deg s^−1^, the average dose per 360° ω scan was estimated to be 0.25 MGy. The coordinates and structure factors of Cas9–RNA–DNA have been deposited in the PDB under accession code 5fq5.

#### 2.1.3. T_2_R-TTL   

Rod-shaped *P*2_1_2_1_2_1_ T_2_R-TTL crystals measuring ∼700 × 100 × 100 µm (Supplementary Fig. S1*c*) were grown, cryoprotected and snap-cooled in liquid nitrogen as described elsewhere (Prota, Bargsten *et al.*, 2013 ▸). Reproducible mounting and cryoprotection was critical as T_2_R-TTL crystals are extremely sensitive to variation of these conditions (Weinert *et al.*, 2015 ▸; PDB entry 4wbn). Of the 20 crystals screened, only three yielded data suitable for merging based on statistical indicators such at *R*_meas_ and 〈*I*/σ(*I*)〉 (Karplus & Diederichs, 2015 ▸). The three crystals diffracted to 2.3 Å resolution. From each crystal, data were collected at two different positions that were well separated. At the first position, a single high-dose 360° ω scan, estimated at ∼4 MGy, was collected (Table 1 ▸ and Supplementary Data; T_2_R-TTL high dose, low multiplicity, single orientation). At the second position, eight low-dose 720° ω scans, corresponding to the same accumulated dose of ∼4 MGy (0.25 MGy per 360° ω scan), were collected: seven at different χ increments from 0 to 30° at constant φ and an additional ω scan at χ = 10 or 15° and φ = 90° (Table 1 ▸ and Supplementary Data; T_2_R-TTL low dose, high multiplicity, multi-orientation).

### 2.2. X-ray fluorescence measurements   

X-ray fluorescence (XRF) analysis was performed on the PHOENIX beamline at the Swiss Light Source at an incident radiation energy of 4.1 keV, which is above the Ca *K* edge (4.038 keV) and not currently accessible at most MX beamlines. The crystal was attached directly to carbon tape and loaded into a 10^−4^ Pa vacuum chamber. The energy-dispersive X-ray fluorescence spectrum was recorded at room temperature using a single-element solid-state detector (from Roentec) with 180 eV energy resolution. The X-ray beam measuring 100 × 100 µm was directed at the center of the crystal. There was no indication of beam damage over the 120 s collection time. The XRF spectrum was fitted using *PyMca* (Solé *et al.*, 2007 ▸) to extract the elemental composition of the probed crystal volume. The software provides the elemental composition in terms of single peaks corresponding to different atomic energy-level transitions using Gaussian shape emission lines and energy-dependent photoelectric cross-sections.

## 3. Results and discussion   

### 3.1. Native SAD phasing   

#### 3.1.1. DgkA   

**Figure 1:**
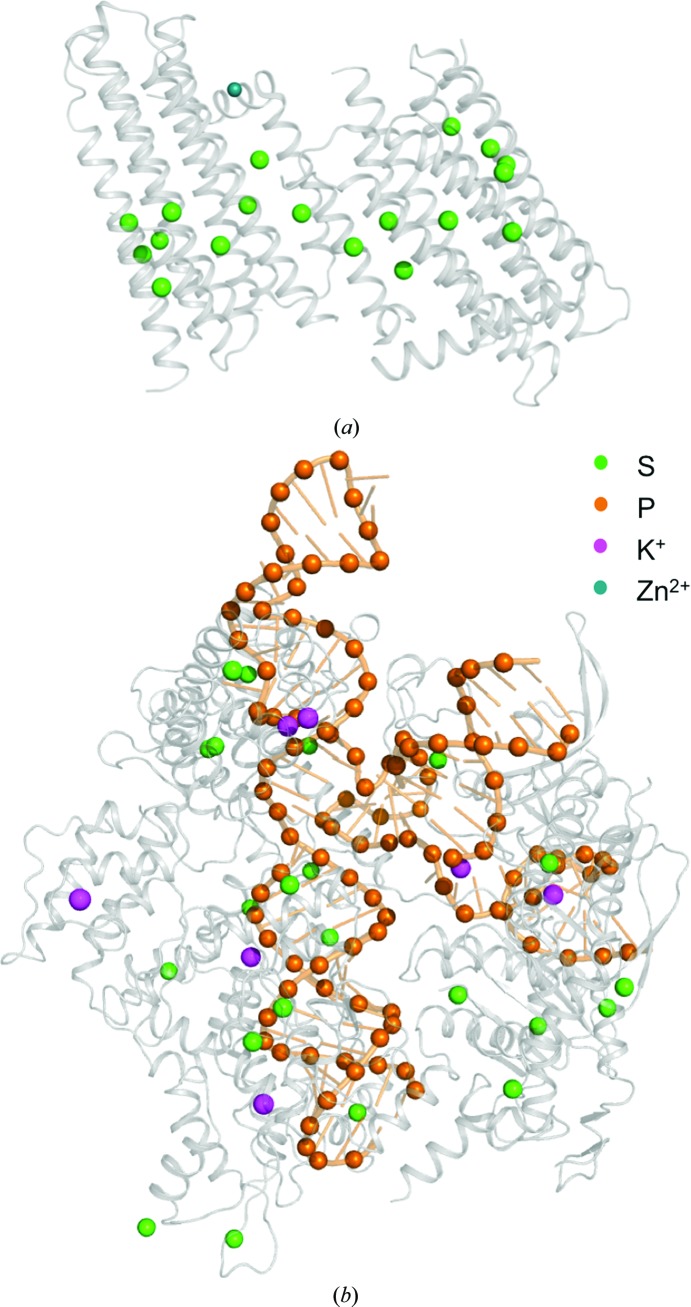
Native SAD structures. (*a*) DgkA and (*b*) Cas9–RNA–DNA. The proteins are shown as grey cartoons and the nucleic acid backbones are coloured orange. Anomalous scatterers are depicted as coloured spheres.

**Figure 2:**
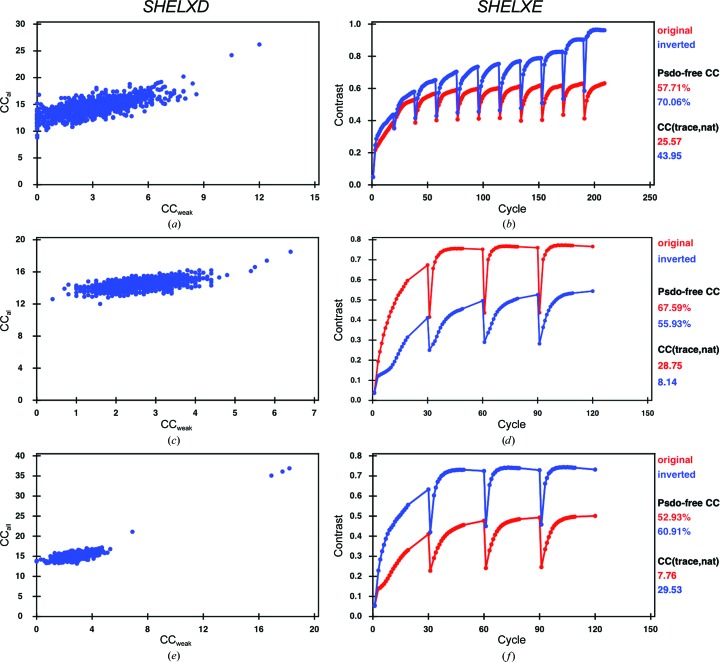
*SHELX* output. (*a*) *SHELXD* substructure-determination output for DgkA for 1000 trials. (*b*) *SHELXE* output for DgkA after ten cycles of auto-tracing. (*c*) *SHELXD* substructure-determination output for Cas9 for 1000 trials. (*d*) *SHELXE* output for Cas9 after three cycles of auto-tracing. (*e*) *SHELXD* substructure determination for T_2_R-TTL with 1000 trials. (*f*) Three cycles of chain tracing with phasing and density modification in *SHELXE* for T_2_R-TTL.

**Figure 3:**
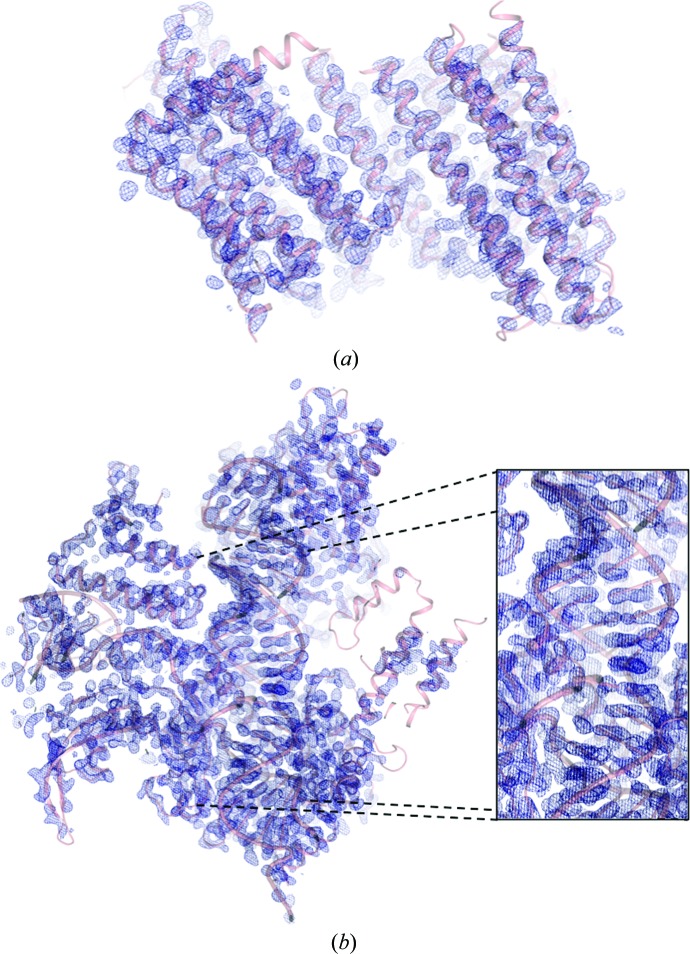
Experimental maps after *SHELXE* phasing and density modification, contoured at 2σ. (*a*) DgkA map. (*b*) Cas9–RNA–DNA map.

The integral membrane diacylglycerol kinase DgkA has two trimers in the asymmetric unit representing 6 × 130 residues with a total molecular weight of 84 kDa. The substructure has 6 × 2 methionines, 6 × 1 cysteines and a zinc ion (Fig. 1 ▸ *a*), corresponding to a theoretical Bijvoet ratio of 1.1% at 6 keV. Previous SAD and MAD attempts at the Zn absorption edge were unsuccessful. The structure was originally solved by Se-SAD phasing at the Se peak using crystals diffracting to 2.05 Å resolution (Li *et al.*, 2013 ▸). The crystals used in the current study only diffracted to 2.6 Å resolution (see §[Sec sec2.1.1]2.1.1). Data were collected both with and without multiple crystal orientations (Table 1 ▸). Substructure identification using *SHELXD* was successful with data cut at 4 Å resolution and with *E* values above 1.5; 14 sites out of 19 were identified, 11 of them correctly as determined using *SITCOM* with reference PDB entry 3ze3 (Li *et al.*, 2013 ▸). The single correct solution obtained after 1000 *SHELXD* attempts had CC_all_ and CC_weak_ values of 26.2 and 12.0, respectively (Fig. 2 ▸ *a*). The correct hand was identified by density modification in *SHELXE*. Auto-tracing with a search for α-helices resulted in 364 residues (out of 780) built after ten cycles (Fig. 2 ▸ *b*), showing a good cross-correlation to the data of 43.95%. The resulting electron-density map (Fig. 3 ▸ *a*) showed a model–map cross-correlation of the *SHELXE* map to the final model of 66.8%.

Analysis of the substructure data exemplifies the weakness of the anomalous data, mainly owing to the disordered Met66 and Cys41 in chains *E* and *F*. The experimental Bijvoet ratio of 1% for this sample was therefore lower than the expected theoretical value of 1.1%.

#### 3.1.2. Cas9–RNA–DNA   

The CRISPR-associated protein Cas9 is an RNA-guided endonuclease that has been repurposed for genome editing and gene-expression control. The Cas9–RNA–DNA complex, which crystallizes in the low-symmetry space group *C*2, was initially solved with SAD phases obtained from selenomethionine and iridium derivatives (Anders *et al.*, 2014 ▸). For native SAD phasing experiments, we used crystals of the Cas9–sgRNA–target DNA complex in which the complementary DNA strand contains mismatches to the guide RNA at positions 1–3 (see §[Sec sec2.1.2]2.1.2). The asymmetric unit contains 1371 amino acids, 83 RNA nucleotides and 39 DNA nucleotides (total molecular weight ∼200 kDa), with 24 S atoms and 120 P atoms (Fig. 1 ▸ *b*), which comprises the largest substructure solved with native SAD phasing to date. In this case, data were collected from three native crystals diffracting to ∼2.2 Å resolution in multiple orientations at a wavelength of 2.066 Å and the data were merged in an initial attempt at locating sulfur and phosphorus positions from the anomalous peaks (Table 1 ▸). The substructure solution could be obtained with 1000 *SHELXD* trials using a high-resolution cutoff of 2.6 Å in a search for 65 sites, excluding data with *E* values below 1.2 (Fig. 2 ▸ *c*). The program initially identified 45 correct sites out of a total of 150 as determined using *SITCOM* with reference PDB entry 4un5 (Anders *et al.*, 2014 ▸). *SHELXE* substructure refinement completed the substructure to 114 sites, resulting in a very clear hand separation (Fig. 2 ▸ *d*). Three cycles of chain tracing with a search for α-helices resulted in a readily interpretable map (Fig. 3 ▸ *b*) building a C_α_ chain of 1060 residues (out of 1372) after three cycles (Fig. 2 ▸ *b*) with a cross-correlation of 28.75% to the data. The model–map cross-correlation of the *SHELXE* map to the final model was 68.0%.

**Figure 4:**
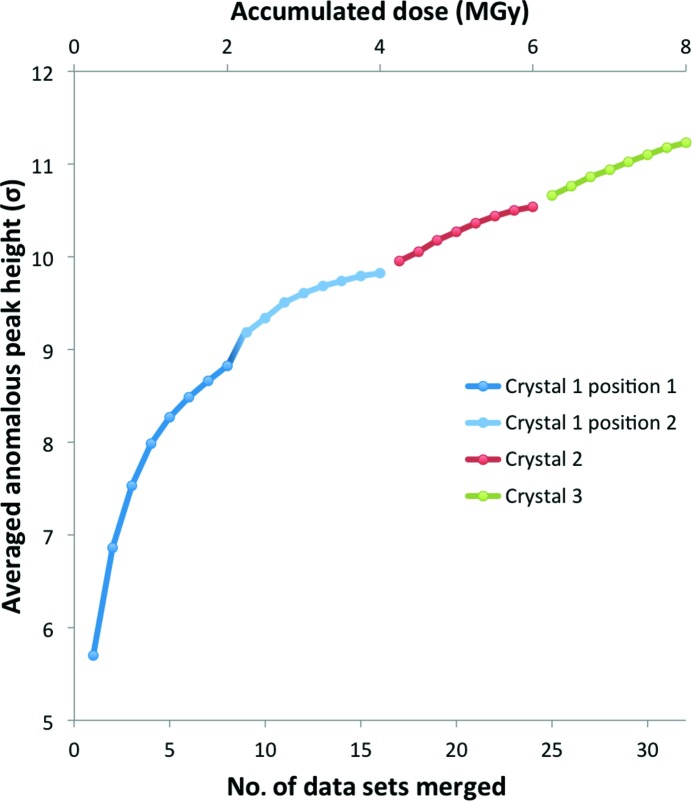
Averaged anomalous peak height of the first 70 anomalous peaks for Cas9–RNA–DNA merged data sets. A total of 3 × 360° data sets were merged from three different crystals. Data from the first crystal were collected at two well separated positions (Supplementary Fig. S1*b*, crystal 1). The X-ray dose (upper *x* axis) accumulated at each position was around 2 MGy.

Substructure determination was only successful using data merged from three crystals collected in multiple orientations. The increase in the anomalous peak height resulting from the merging of new data sets (coming from either new orientations of the same crystal or from additional crystals) is shown in Fig. 4 ▸. We limited the accumulated dose on each crystal (or crystal location) to a conservative ∼2 MGy in order to minimize the effects of radiation damage. Additional dose brought little additional information; in the case of crystal 1, we observed that the gain in anomalous signal was small beyond 2 MGy and plateaued at 4 MGy (Supplementary Fig. S2).

### 3.2. X-ray fluorescence analysis   

**Figure 5:**
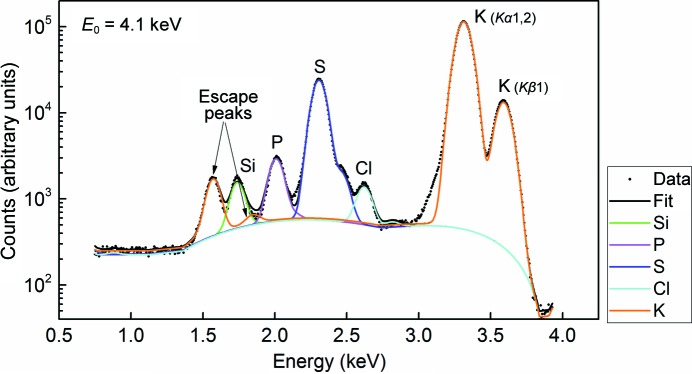
Log scale of the X-ray fluorescence spectrum of a Cas9–RNA–DNA crystal recorded at an incident energy of 4.1 keV. The measured spectrum is plotted as black dots and the fit as black (total) and coloured (for different elements) lines. The main contributions (orange) originate from the potassium *K*α1,2 (*K*–*L*_III_ and *K*–*L*_II_) and *K*β1 (*K*–*M*_III_) characteristic lines. The smaller (orange) peaks between 1.5 and 2 keV represent the K–Si escape peaks and are an artifact of the measurement (Papp & Campbell, 2001 ▸). The Si peak (green line) originates from the crystal holder.

Assigning certain atoms, especially light monoatomic ions, in a crystal structure can be difficult. An added benefit of data collection at low energy is that the anomalous scattering from some of these elements can aid in their correct assignment (Mueller-Dieckmann *et al.*, 2007 ▸). In the case of the Cas9–sgRNA–DNA complex, certain ions originally modelled as Mg^2+^ (PDB entry 4un5) displayed strong anomalous signal (>10σ), suggesting alternative atom assignments. However, it was not possible to unambiguously identify the corresponding element from the anomalous peak heights or from an examination of the chemical architecture of the site. One of the crystals was subsequently used to collect a fluorescence spectrum at an incident energy of 4.1 keV (see §[Sec sec2.2]2.2). The spectrum confirmed the presence of K^+^ (Fig. 5 ▸), which was likely to arise from the crystallization condition (the buffer solution contained 250 m*M* KCl and 300 m*M* KSCN). We therefore reassigned six Mg^2+^ ions as K^+^ ions and found an additional one. Subsequent refinement of the anomalous scattering contribution *f*′′, as implemented in *phenix.refine* (Echols *et al.*, 2014 ▸), gave *f*′′(K^+^) values between 1.58 and 1.72 e, in agreement with the theoretical value of 1.77 e.

### 3.3. Optimal data-collection strategy for challenging native SAD phasing: T_2_R-TTL as a test case   

T_2_R-TTL is a multiprotein/multiligand complex composed of tubulin (T), the microtubule destabilizing- and stathmin-like protein RB3 and the tubulin-modifying enzyme tubulin tyrosine ligase (TTL; Prota, Magiera *et al.*, 2013 ▸). The asymmetric unit, composed of two α-tubulin, two β-tubulin, one RB3, one TTL, two GTP, two GDP and one AMPPNP molecules, consists of 2317 amino acids (total molecular weight of ∼266 kDa) and has a substructure with 118 S, 13 P, three Ca and two Cl sites. Following our previous successful structure solution of T_2_R-TTL by native SAD phasing using a single crystal, multi-orientation data-collection strategy (Weinert *et al.*, 2015 ▸), we decided to use this system to evaluate the benefits of the combined multi-orientation, multi-crystal strategy. T_2_R-TTL is an excellent example of a challenging test case; it has a large asymmetric unit and a large number of scatterers and, as shown below, it forms crystals of variable quality.

Using multiple native SAD data sets measured from a single T_2_R-TTL crystal (see §[Sec sec2.1.3]2.1.3 and Supplementary Data), it was not possible to determine the substructure. This is likely because the quality of the crystals used in the current work was less than that observed in the original study (Weinert *et al.*, 2015 ▸). A substructure solution with *SHELXD* could only be obtained upon merging data sets from three different crystals each measured in eight different orientations (Table 1 ▸ and Supplementary Data) in a search for 75 sites using a resolution cutoff of 3.3 Å and with *E* values above 1.5 (Fig. 2 ▸ *e*). The successful *SHELXD* sub­structure solution had a CC_all_ and a CC_weak_ of 36.9 and 18.2, respectively. The number of correct sites, as determined using *SITCOM* with reference PDB entry 4wbn, was 86 and this increased to 111 using substructure refinement in *SHELXE*. Density modification resulted in a clear separation of hands (Fig. 2 ▸ *f*). Three cycles of chain tracing with a search for α-helices resulted in a readily interpretable map, building a C_α_ chain of 1669 residues (out of 2319) after three cycles with a cross-correlation of 28.75% to the data. The model–map cross-correlation of the *SHELXE* map to the final model was 78.5%.

**Figure 6:**
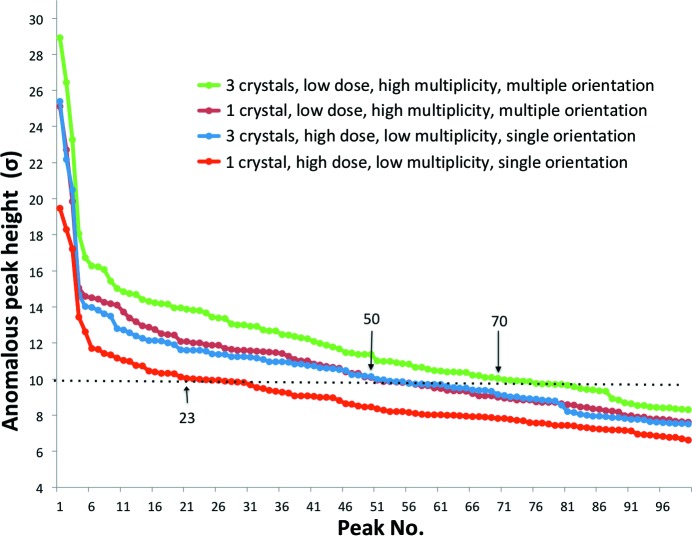
Comparison of anomalous peak heights for various data-collection strategies. The anomalous peak heights for the first 100 peaks are shown for four data-collection strategies for T_2_R-TTL data sets: (i) high dose, low multiplicity, single orientation at a single crystal location (red), (ii) low dose, high multiplicity, multiple orientation at a single crystal location (brick), (iii) merging high-dose, single-orientation data from three crystals (blue) and (iv) merging low-dose, multiple-orientation data from three crystals (green). For each crystal, data were collected with the same dose at two well separated positions. The dotted line marks the 10σ threshold. The arrows point to the last peak above 10σ and show the number of sites above this threshold.

The merit of combining low-dose data from multiple crystals in multiple orientations, which was readily observed in the merging statistics (see Supplementary Data), was assessed by comparing the average anomalous peak heights for four data-collection strategies: (i) high dose, single orientation from a single crystal; (ii) low dose, multiple orientation from a single crystal; (iii) merging high-dose, single-orientation data from three crystals; and (iv) merging low-dose, multiple-orientation data from three crystals (Fig. 6 ▸). A single-orientation scan at high dose gave only 23 anomalous peaks above 10σ, which was insufficient for successful substructure solution. Despite a threefold higher accumulated dose, merging high-dose data from three different crystals resulted in 50 anomalous sites above 10σ, similar to merging low-dose, multiple-orientation data from a single crystal. However, this was still insufficient for successful substructure solution. The substructure search was only successful when low-dose, multiple-orientation data sets from three crystals were merged, yielding 70 anomalous sites above 10σ. This is consistent with our previous study, in which substructure solution and hence native SAD phasing were successful by merging low-dose, multiple-orientation scans from a single crystal, giving 80 anomalous sites above 10σ (Weinert *et al.*, 2015 ▸). This is also consistent with a study showing that substructure searches tend to be successful when mean peak heights in the anomalous difference map are above a certain threshold (Bunkóczi *et al.*, 2015 ▸); for T_2_R-TTL crystals, this threshold is around 10σ (dashed line in Fig. 6 ▸).

Taken together, there is a clear benefit in combining the two data-collection strategies: (i) a low-dose, multiple-orientation approach efficiently extracts as much anomalous data as possible while minimizing radiation damage and (ii) if the anomalous signal from a single crystal sample is not sufficient for successful phasing, data sets from multiple statistically equivalent crystals can be merged. Combining the two approaches means that fewer crystal samples are required. In addition, for the same accumulated dose, there is no difference in diffraction resolution between data sets collected using a high-dose, single-orientation strategy and merged data sets collected using a low-dose, multiple-orientation strategy (Table 1 ▸ and Supplementary Data).

## 4. Conclusion and outlook   

Native SAD could be considered the ‘ideal’ *de novo* phasing method for macromolecules because it relies solely on information from atoms that are naturally present in the sample. The method is readily applicable to challenging cases. Accordingly, there is every reason to try native SAD first with macromolecules that contain a sufficient number of naturally occurring anomalous scatterers. Here, we show that multi-crystal averaging of data sets used in conjunction with a low-dose, multi-orientation strategy is beneficial in difficult cases where data from one crystal are insufficient. Further, low-dose data collection in multiple orientations means that fewer crystal specimens are needed to provide the required data accuracy.

That said, this combined approach is still in its infancy and there is room for improvement in order to make native SAD more broadly applicable and more generally accepted, especially in cases of low resolution, which are currently dominated by heavy-atom phasing methods. In addition, collecting multiple 360° scans on multiple crystals, as detailed here, takes more time than single-orientation high-dose scans and may be unappealing in situations where synchrotron beamtime is limited. Nevertheless, advances in data-acquisition hardware, in particular detector instrumentation, will rapidly improve the situation. Furthermore, the method described here lends itself to automation.

As the frontiers of crystallography continue to expand, fundamental techniques for solving the phase problem will continue to evolve. In particular, new methods such as serial crystallography have presented new challenges that require the development of tools for obtaining the most accurate measurements. Data collection at energies below 6 keV should boost the anomalous signal, but presents technical challenges, especially air absorption and scattering, sample absorption and detector geometry. These are currently being investigated at operational beamlines such as BL-1A at the Photon Factory (see Rose *et al.*, 2015 ▸), as well as upcoming beamlines such as I23 at Diamond Light Source (Allan *et al.*, 2015 ▸) and LAX at NSLS-II (Hendrickson, 2014 ▸).

## Supplementary Material

Supporting information. DOI: 10.1107/S2059798315024110/ba5243sup1.pdf

## Figures and Tables

**Table 1:** Summary of data collection and statistics Values in parentheses are for the last shell.

Data set	DgkA	Cas9–RNA–DNA	T_2_R-TTL (high dose, low multiplicity, single orientation)†	T_2_R-TTL (low dose, high multiplicity, multi-orientation)†
No. of crystals	3	3	3	3
Total oscillation (χ/φ orientations)	Crystal 1: 10 × 360°, 0.1 s, 0.1° (χ = 0°)	Crystal 1 p1‡: 8 × 360°, 0.1 s, 0.2° (χ = 0°, χ = 10°, χ = 20°, χ = 30°, χ = 30°/φ = 180°, χ = 20°/φ = 180°, χ = 10°/φ = 180°, χ = 5°/φ = 180°)	Crystal 1 p1‡: 360°, 1.6 s, 0.2°	Crystal 1 p2‡: 8 × 720°, 0.1 s, 0.2° (χ = 0°, χ = 5°, χ = 10°, χ = 15°, χ = 20°, χ = 25°, χ = 30°, χ = 10°/φ = 90°)
	Crystal 2: 6 × 360°, 0.2 s, 0.2° (χ = 0°), 2 × 360°, 0.2 s, 0.2° (a* aligned)	Crystal 1 p2‡: 8 × 360°, 0.1 s, 0.2° (χ = 5°/φ = 45°, χ = 15°/φ = 45°, χ = 25°/φ = 45°, χ = 13°, χ = 13°/φ = 45°, χ = 18°, χ = 18°/φ = 45°, χ = 23°/φ = 45°)	Crystal 2 p1‡: 360°, 1.6 s, 0.2°	Crystal 2 p2‡: 8 × 720°, 0.1 s, 0.2° (χ = 0°, χ = 10°, χ = 20°, χ = 30°, χ = 25°, χ = 15°, χ = 5°, χ = 15°/φ = 90°)
	Crystal 3: 4 × 360°, 0.1 s, 0.1° (χ = 0°), 2 × 360°, 0.2 s, 0.2° (χ = 10°), 2 × 720°, 0.1 s, 0.1° (χ = 20°, χ = 30°)	Crystal 2: 8 × 360°, 0.1 s, 0.1° (χ = 0°, χ = 5°, χ = 10°, χ = 15°, χ = 20°, χ = 25°, χ = 30°, χ = 35°)	Crystal 3 p1‡: 360°, 1.6 s, 0.2°	Crystal 3 p2‡: 8 × 720°, 0.1 s, 0.2° (χ = 0°, χ = 10°, χ = 20°, χ = 30°, χ = 25°, χ = 15°, χ = 5°, χ = 10°/φ = 90°)
		Crystal 3: 8 × 360°, 0.1 s, 0.2° (χ = 0°, χ = 10°, χ = 20°, χ = 30°, χ = 30°/φ = 180°, χ = 20°/φ = 180°, χ = 10°/φ = 180°, χ = 5°/φ = 180°)		
Resolution (Å)	50.0–2.6 (2.67–2.60)	50–2.2 (2.30–2.20)	50–2.3 (2.39–2.30)	50–2.3 (2.39–2.30)
Space group	*P*2_1_2_1_2_1_	*C*2	*P*2_1_2_1_2_1_	*P*2_1_2_1_2_1_
Unit-cell parameters (Å, °)	*a* = 75.29, *b* = 91.57, *c* = 143.72	*a* = 177.74, *b* = 67.57, *c* = 188.19, β = 111.32	*a* = 104.88, *b* = 158.68, *c* = 180.04	*a* = 104.86, *b* = 158.57, *c* = 180.31
No. of reflections	8396768 (235461)	16157127 (388880)	4330651 (206334)	69786479 (3327104)
No. of unique reflections	29385 (2108)	205825 (24766)	255989 (27045)	258112 (28073)
Multiplicity§ (°)	142.9 (55.84)	156.7 (31.4)	33.8 (15.3)	540.7 (237.0)
Completeness (%)	99.6 (96.5)	99.3 (95.8)	99.2 (96.6)	100.0 (100.0)
〈*I*/σ(*I*)〉	36.46 (4.18)	30.38 (1.88)	18.70 (1.23)	39.59 (1.19)
*R*_meas_ (%)	22.1 (113.1)	14.1 (135.0)	11.1 (162.3)	25.5 (539.5)
*R*_p.i.m._ (%)	1.8 (15.1)	1.1 (24.1)	1.9 (41.5)	1.1 (35.0)
CC_1/2_ (%)	100.0 (90.8)	100.0 (62.1)	99.8 (54.2)	100.0 (52.4)
Δ*F*/σ(Δ*F*)	1.52	1.477	1.13	2.38
CC_ano_ (%)	43	44	34	63
Mosaicity (°)	0.09/0.19/0.26	0.10/0.16/0.07	0.10/0.10/0.10	0.10/0.10/0.10

† See Supplementary Data for *XDS*/*XSCALE* processing statistics of both merged and individual data sets.

‡ For crystals that were translated during measurement, the different positions are labelled p1 and p2.

§ Friedel pairs are counted as merged reflections.
