# Supplementary material for: Data-collection strategy for challenging native SAD phasing
Source: Acta Crystallogr D Struct Biol. 2016 Mar 1;72(Pt 3):421–9. doi: 10.1107/S2059798315024110 (PMC4784673; doi:10.1107/S2059798315024110)
Supplement: Supplementary file 1 [file d-72-00421-sup1.pdf]

STRUCTURAL
BIOLOGY

Volume 72 (2016)

Supporting information for article:

Data-collection strategy for challenging native SAD phasing

Vincent Olieric, Tobias Weinert, Aaron D. Finke, Carolin Anders, Dianfan Li, Natacha Olieric, Camelia N. Borca, Michel O. Steinmetz, Martin Caffrey, Martin Jinek and Meitian Wang

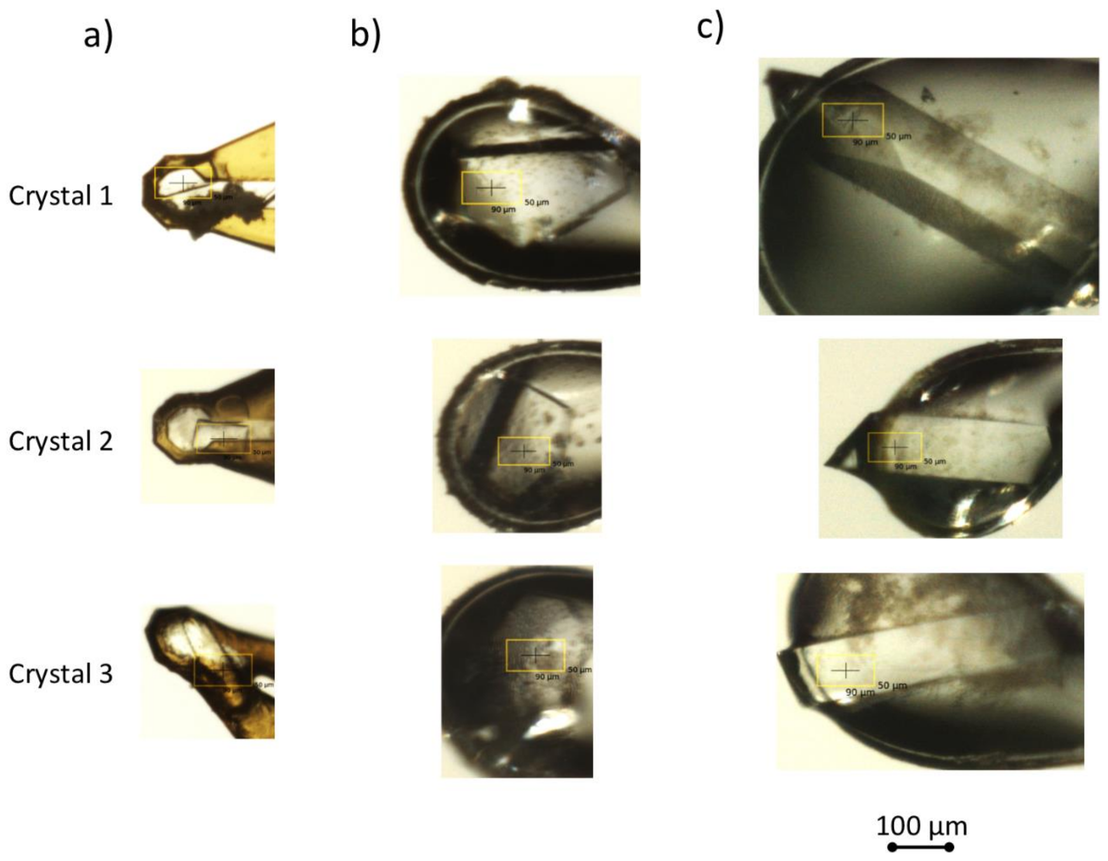

Figure S1 Crystal pictures. (a) DgkA crystals 1-2-3. (b) Cas9–RNA–DNA crystals 1-2-3. (c) T_2R-TTL crystals 1-2-3. The yellow rectangle represents the 90 × 50 µm^2 sized X-ray beam.

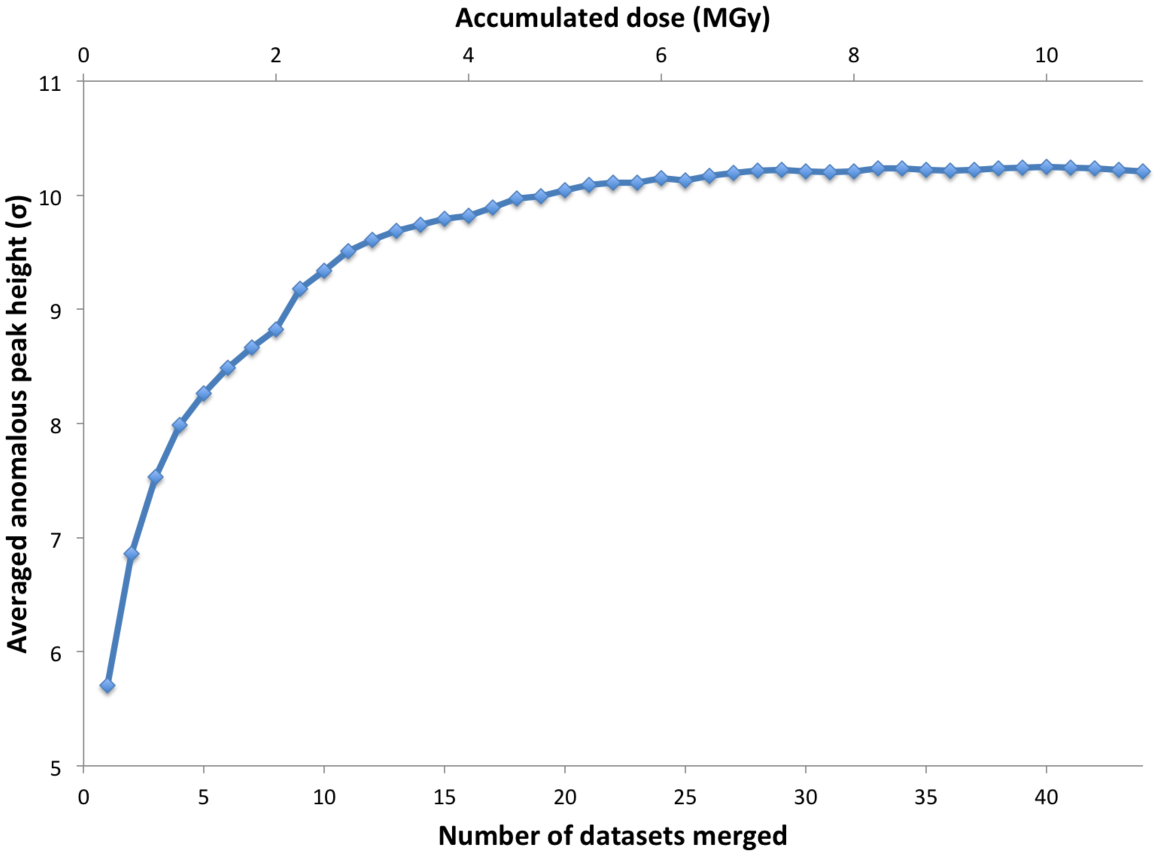

Figure S2 Averaged anomalous peak height of the first 70 peaks of the Cas9–RNA–DNA merged data set. The dose was distributed over Crystal 1 (Supplementary Figure 1b) by changing the position of the crystal in the beam and by adjusting the PRIGo χ/φ reorientations after each 360° of data collection (Table 1)

Supplementary Data XDS/XSCALE processing/merging statistics of T_2R-TTL data sets.

T2R-TTL (high dose, low multiplicity, single orientation)
Crystals 1,2,3 p1 XSCALE merged data (see individual data sets below)

SUBSET OF INTENSITY DATA WITH SIGNAL/NOISE >= -3.0 AS FUNCTION OF RESOLUTION

RESOLUTION LIMIT	NUMBER OF REFLECTIONS OBSERVED	UNIQUE	POSSIBLE	COMPLETENESS OF DATA	R-FACTOR observed	R-FACTOR expected	COMPARED	I/SIGMA	R-meas	CC(1/2)	Anomal Corr	SigAno	Nano
20.00	7277	372	400	93.0%	5.0%	5.5%	7277	60.13	5.1%	98.6*	81*	2.730	134
10.00	60571	2750	2750	100.0%	3.8%	5.6%	60571	63.54	3.9%	100.0*	83*	2.270	1187
6.00	229600	11427	11427	100.0%	4.8%	5.8%	229600	52.76	4.9%	100.0*	69*	1.989	5266
3.00	2002582	101694	101695	100.0%	8.6%	8.5%	2002580	32.77	8.8%	99.9*	15*	1.010	48755
2.88	277633	15149	15152	100.0%	28.2%	30.8%	277631	10.87	29.0%	99.0*	-2	0.693	7326
2.76	313771	17871	17875	100.0%	38.2%	42.5%	313770	7.85	39.3%	98.3*	-4	0.673	8672
2.65	334217	19397	19403	100.0%	52.0%	58.4%	334107	5.79	53.5%	96.6*	-5	0.650	9336
2.55	351373	20489	20575	99.6%	62.6%	72.6%	351139	4.60	64.5%	94.5*	-3	0.645	9789
2.47	292291	18700	18996	98.4%	80.5%	95.6%	291983	3.31	83.2%	90.2*	-5	0.622	8907
2.39	255002	21095	21692	97.2%	104.0%	125.4%	254518	2.26	108.5%	81.9*	-1	0.621	9949
2.30	206334	27045	27989	96.6%	151.3%	184.3%	205310	1.23	162.3%	54.2*	-2	0.591	12542
total	4330651	255989	257954	99.2%	10.8%	11.5%	4328486	18.70	11.1%	99.8*	11*	0.863	121863

-

T2R-TTL (low dose, high multiplicity, multi-orientation)
Crystals 1,2,3 p2 XSCALE merged data (see individual data sets below)

SUBSET OF INTENSITY DATA WITH SIGNAL/NOISE >= -3.0 AS FUNCTION OF RESOLUTION

RESOLUTION LIMIT	NUMBER OF REFLECTIONS OBSERVED	UNIQUE	POSSIBLE	COMPLETENESS OF DATA	R-FACTOR observed	R-FACTOR expected	COMPARED	I/SIGMA	R-meas	CC(1/2)	Anomal Corr	SigAno	Nano
20.00	115957	372	400	93.0%	6.0%	5.7%	115957	221.91	6.0%	100.0*	98*	9.139	134
10.00	1007385	2752	2752	100.0%	4.5%	6.0%	1007385	224.73	4.5%	100.0*	97*	7.092	1188
6.00	3796112	11429	11429	100.0%	6.8%	7.5%	3796112	153.88	6.8%	100.0*	91*	4.766	5265
3.00	32542854	101771	101775	100.0%	19.2%	17.6%	32542854	69.43	19.3%	100.0*	38*	1.639	48788
2.88	4430340	15148	15152	100.0%	108.8%	109.1%	4430340	12.75	109.0%	99.1*	5	0.816	7330
2.76	4995505	17887	17892	100.0%	150.7%	153.8%	4995505	8.63	151.0%	98.4*	1	0.764	8676
2.65	5282998	19414	19422	100.0%	202.7%	209.4%	5282998	5.89	203.1%	96.4*	1	0.742	9423
2.55	5548790	20582	20584	100.0%	248.2%	259.9%	5548790	4.44	248.6%	93.8*	1	0.720	10002
2.47	4655566	18992	18995	100.0%	316.4%	335.4%	4655566	3.11	317.1%	88.4*	2	0.701	9242
2.39	4083868	21692	21693	100.0%	396.0%	423.0%	4083868	2.11	397.0%	76.2*	0	0.684	10563
2.30	3327104	28073	28074	100.0%	537.3%	578.7%	3327104	1.19	539.5%	52.4*	1	0.662	13682
total	69786479	258112	258168	100.0%	25.5%	25.2%	69786479	39.59	25.6%	100.0*	20*	1.322	124293

T2R-TTL (high dose, low multiplicity, single orientation)
Individual data sets - XDS CORRECT

Crystal 1 p1 360° 1.6s 0.2°

SUBSET OF INTENSITY DATA WITH SIGNAL/NOISE >= -3.0 AS FUNCTION OF RESOLUTION

RESOLUTION LIMIT	NUMBER OF REFLECTIONS OBSERVED	UNIQUE	POSSIBLE	COMPLETENESS OF DATA	R-FACTOR observed	R-FACTOR expected	COMPARED	I/SIGMA	R-meas	CC(1/2)	Anomal Corr	SigAno	Nano
6.35	84037	12155	12186	99.7%	3.9%	3.8%	84036	43.64	4.2%	99.9*	60*	1.744	5468
4.51	149986	21773	21777	100.0%	4.5%	4.3%	149982	37.20	4.9%	99.8*	39*	1.297	10273
3.69	189312	28216	28219	100.0%	5.0%	4.9%	189311	31.80	5.4%	99.8*	17*	0.997	13496
3.20	210584	33299	33314	100.0%	8.4%	8.5%	210546	18.38	9.2%	99.6*	3	0.826	16008
2.86	228208	37815	37826	100.0%	16.7%	17.9%	228148	9.07	18.2%	98.6*	-2	0.723	18247
2.61	233302	41709	41771	99.9%	40.7%	43.7%	233017	3.83	44.8%	92.7*	-4	0.685	19999
2.42	233723	44570	45329	98.3%	76.1%	86.1%	232501	1.87	84.3%	75.7*	-3	0.656	20863
2.26	120269	40196	48790	82.4%	145.3%	172.4%	112014	0.58	172.6%	32.5*	-2	0.591	13788
2.13	27933	18759	51889	36.2%	228.7%	286.0%	15727	0.15	301.1%	11.6*	4	0.542	1882
total	1477354	278492	321101	86.7%	8.0%	8.2%	1455282	12.43	8.7%	99.8*	8	0.827	120024

Crystal 2 p1 360° 1.6s 0.2°

SUBSET OF INTENSITY DATA WITH SIGNAL/NOISE >= -3.0 AS FUNCTION OF RESOLUTION

RESOLUTION LIMIT	NUMBER OF REFLECTIONS OBSERVED	UNIQUE	POSSIBLE	COMPLETENESS OF DATA	R-FACTOR observed	R-FACTOR expected	COMPARED	I/SIGMA	R-meas	CC(1/2)	Anomal Corr	SigAno	Nano
6.36	83737	12225	12254	99.8%	2.9%	3.1%	83736	53.83	3.2%	99.9*	69*	1.989	5501
4.51	148734	21905	21913	100.0%	3.9%	3.6%	148726	43.90	4.2%	99.9*	48*	1.480	10328
3.69	188934	28345	28352	100.0%	4.5%	4.2%	188923	36.51	4.9%	99.9*	24*	1.115	13552
3.20	219410	33527	33546	99.9%	8.1%	7.9%	219317	20.33	8.8%	99.7*	6	0.879	16064
2.86	245373	37851	38006	99.6%	16.2%	17.4%	244800	9.82	17.6%	98.8*	0	0.741	17958
2.61	245187	40723	41921	97.1%	36.4%	40.2%	244693	4.36	39.8%	94.2*	-5	0.668	19315
2.42	240407	42135	45724	92.2%	59.4%	68.5%	239424	2.42	65.4%	82.9*	-3	0.645	19630
2.26	114236	37715	48932	77.1%	103.3%	121.5%	108642	0.92	123.6%	49.1*	-2	0.612	15228
2.13	31187	16664	52188	31.9%	155.1%	189.1%	24937	0.40	204.4%	19.9*	1	0.578	3170
total	1517206	271091	322836	84.0%	7.0%	7.3%	1503198	14.86	7.7%	99.9*	12*	0.874	120746

Crystal 3 p1 360° 1.6s 0.2°

SUBSET OF INTENSITY DATA WITH SIGNAL/NOISE >= -3.0 AS FUNCTION OF RESOLUTION

RESOLUTION LIMIT	NUMBER OF REFLECTIONS OBSERVED	UNIQUE	POSSIBLE	COMPLETENESS OF DATA	R-FACTOR observed	R-FACTOR expected	COMPARED	I/SIGMA	R-meas	CC(1/2)	Anomal Corr	SigAno	Nano
6.29	87714	12631	12663	99.7%	3.2%	3.2%	87713	49.79	3.4%	99.9*	65*	1.843	5691
4.47	153888	22633	22645	99.9%	4.5%	4.1%	153876	38.40	4.8%	99.9*	38*	1.330	10683
3.65	195360	29248	29255	100.0%	6.0%	5.5%	195350	28.99	6.5%	99.8*	15*	1.012	13986
3.17	225189	34537	34563	99.9%	13.0%	12.8%	225073	13.75	14.2%	99.3*	2	0.829	16552
2.83	246226	39036	39273	99.4%	30.2%	32.1%	245835	5.75	32.9%	96.4*	-2	0.718	18603
2.59	251655	41794	43441	96.2%	67.7%	73.9%	250732	2.40	74.1%	84.0*	-3	0.658	19594
2.39	211034	43627	47148	92.5%	119.1%	136.1%	209030	1.09	133.3%	54.0*	-3	0.613	19848
2.24	114437	40507	50589	80.1%	222.3%	264.0%	107035	0.40	269.6%	16.7*	-1	0.553	15002
2.11	26331	17699	53882	32.8%	362.6%	443.4%	15178	0.17	483.9%	2.3	2	0.537	1829
total	1511834	281712	333459	84.5%	10.0%	10.3%	1489822	11.40	11.0%	99.8*	8	0.823	121788

T2R-TTL (low dose, high multiplicity, multi-orientation)
Individual data sets - XDS CORRECT

Crystal 1 p2 720° 0.1s 0.2° χ 0°

SUBSET OF INTENSITY DATA WITH SIGNAL/NOISE >= -3.0 AS FUNCTION OF RESOLUTION

RESOLUTION LIMIT	NUMBER OF REFLECTIONS OBSERVED	UNIQUE	POSSIBLE	COMPLETENESS OF DATA	R-FACTOR observed	R-FACTOR expected	COMPARED	I/SIGMA	R-meas	CC(1/2)	Anomal Corr	SigAno	Nano
6.37	173679	12181	12210	99.8%	4.0%	3.9%	173679	57.30	4.1%	100.0*	65*	1.945	5481
4.52	307026	21834	21834	100.0%	6.7%	6.4%	307026	36.75	7.0%	99.9*	35*	1.271	10303
3.70	382196	28280	28281	100.0%	8.9%	8.5%	382196	27.77	9.3%	99.8*	14*	0.998	13532
3.20	421110	33424	33425	100.0%	20.8%	20.4%	421110	12.10	21.7%	99.0*	5	0.857	16096
2.87	461129	37870	37878	100.0%	46.8%	46.8%	461129	5.20	48.8%	95.2*	0	0.786	18326
2.62	460250	41816	41862	99.9%	102.9%	104.8%	460249	2.18	107.9%	77.2*	1	0.729	20291
2.42	447518	44807	45500	98.5%	175.6%	182.3%	447513	1.13	185.1%	45.3*	1	0.682	21712
2.27	210222	39358	48804	80.6%	273.7%	289.2%	210172	0.45	304.6%	12.3*	3	0.630	18157
2.14	49027	17851	52016	34.3%	350.3%	373.9%	48921	0.22	443.0%	4.2	3	0.603	5838
total	2912157	277421	321810	86.2%	17.6%	17.6%	2911995	11.00	18.5%	99.5*	8	0.848	129736

Crystal 1 p2 720° 0.1s 0.2° χ 5°

SUBSET OF INTENSITY DATA WITH SIGNAL/NOISE >= -3.0 AS FUNCTION OF RESOLUTION

RESOLUTION LIMIT	NUMBER OF REFLECTIONS OBSERVED	UNIQUE	POSSIBLE	COMPLETENESS OF DATA	R-FACTOR observed	R-FACTOR expected	COMPARED	I/SIGMA	R-meas	CC(1/2)	Anomal Corr	SigAno	Nano
6.37	173835	12183	12212	99.8%	4.0%	3.9%	173835	56.43	4.2%	100.0*	64*	1.945	5482
4.52	308066	21853	21853	100.0%	7.0%	6.6%	308066	35.80	7.3%	99.9*	31*	1.234	10312
3.70	384063	28292	28293	100.0%	9.3%	8.9%	384063	26.94	9.7%	99.8*	14*	1.003	13537
3.20	415818	33447	33450	100.0%	22.1%	21.7%	415818	11.47	23.0%	98.9*	5	0.855	16107
2.87	450543	37875	37888	100.0%	50.2%	50.2%	450543	4.81	52.4%	94.5*	2	0.779	18330
2.62	454065	41826	41864	99.9%	110.6%	112.9%	454064	2.00	116.1%	74.8*	1	0.726	20295
2.42	438935	45079	45552	99.0%	187.4%	195.0%	438932	1.03	197.9%	41.4*	0	0.672	21878
2.27	199858	36079	48808	73.9%	289.6%	306.5%	199823	0.42	320.9%	11.2*	1	0.618	16579
2.14	48554	16602	52056	31.9%	365.8%	388.7%	48475	0.21	454.6%	4.6	2	0.587	5684
total	2873737	273236	321976	84.9%	18.2%	18.2%	2873619	10.78	19.2%	99.5*	8	0.843	128204

Crystal 1 p2 720° 0.1s 0.2° χ 10°

SUBSET OF INTENSITY DATA WITH SIGNAL/NOISE >= -3.0 AS FUNCTION OF RESOLUTION

RESOLUTION LIMIT	NUMBER OF REFLECTIONS OBSERVED	UNIQUE	POSSIBLE	COMPLETENESS OF DATA	R-FACTOR observed	R-FACTOR expected	COMPARED	I/SIGMA	R-meas	CC(1/2)	Anomal Corr	SigAno	Nano
6.37	174060	12192	12222	99.8%	4.1%	3.9%	174060	56.30	4.2%	100.0*	62*	1.932	5486
4.52	308598	21879	21879	100.0%	7.2%	6.7%	308598	35.32	7.4%	99.9*	31*	1.215	10324
3.70	386348	28310	28310	100.0%	9.5%	9.1%	386348	26.47	9.9%	99.8*	13*	0.992	13545
3.20	405544	33513	33516	100.0%	23.0%	22.5%	405544	10.87	24.0%	98.8*	3	0.849	16139
2.87	444804	37922	37931	100.0%	53.3%	53.3%	444804	4.51	55.7%	93.8*	2	0.783	18349
2.62	451668	41810	41863	99.9%	117.9%	120.5%	451667	1.86	123.8%	72.2*	1	0.715	20287
2.42	440061	45212	45643	99.1%	201.5%	209.6%	440047	0.95	212.8%	38.9*	1	0.671	21962
2.27	200189	33180	48831	67.9%	301.5%	318.7%	200131	0.43	330.9%	11.2*	2	0.621	15550
2.14	49018	14366	52169	27.5%	391.6%	418.0%	48934	0.21	469.7%	3.9	2	0.582	5722
total	2860290	268384	322364	83.3%	18.7%	18.7%	2860133	10.74	19.7%	99.5*	7	0.839	127364

Crystal 1 p2 720° 0.1s 0.2° χ 15°

SUBSET OF INTENSITY DATA WITH SIGNAL/NOISE >= -3.0 AS FUNCTION OF RESOLUTION

RESOLUTION LIMIT	NUMBER OF REFLECTIONS OBSERVED	UNIQUE	POSSIBLE	COMPLETENESS OF DATA	R-FACTOR observed	R-FACTOR expected	COMPARED	I/SIGMA	R-meas	CC(1/2)	Anomal Corr	SigAno	Nano
6.37	173893	12208	12237	99.8%	4.1%	4.0%	173893	55.96	4.3%	100.0*	61*	1.871	5493
4.52	307734	21888	21888	100.0%	7.2%	6.8%	307734	35.02	7.5%	99.9*	31*	1.215	10329
3.70	387707	28337	28337	100.0%	9.7%	9.2%	387707	26.32	10.0%	99.8*	14*	0.992	13559
3.20	407299	33516	33519	100.0%	23.3%	22.8%	407299	10.82	24.3%	98.8*	4	0.850	16140
2.87	441301	37969	37983	100.0%	54.2%	54.3%	441301	4.43	56.7%	93.6*	1	0.781	18376
2.62	449619	41902	41910	100.0%	121.3%	124.4%	449619	1.81	127.4%	71.8*	2	0.723	20342
2.42	438317	45375	45654	99.4%	208.0%	217.3%	438305	0.92	219.7%	38.5*	1	0.667	22042
2.27	197074	32337	48889	66.1%	315.6%	335.6%	196998	0.40	346.1%	11.0*	1	0.608	15218
2.14	46585	13710	52211	26.3%	373.9%	397.9%	46475	0.22	448.2%	4.9	2	0.588	5640
total	2849529	267242	322628	82.8%	18.7%	18.7%	2849331	10.70	19.7%	99.5*	8	0.836	127139

Crystal 1 p2 720° 0.1s 0.2° χ 20°

SUBSET OF INTENSITY DATA WITH SIGNAL/NOISE >= -3.0 AS FUNCTION OF RESOLUTION

RESOLUTION LIMIT	NUMBER OF REFLECTIONS OBSERVED	UNIQUE	POSSIBLE	COMPLETENESS OF DATA	R-FACTOR observed	R-FACTOR expected	COMPARED	I/SIGMA	R-meas	CC(1/2)	Anomal Corr	SigAno	Nano
6.37	173994	12220	12249	99.8%	4.1%	4.0%	173994	55.59	4.3%	100.0*	61*	1.883	5499
4.52	306833	21933	21933	100.0%	7.2%	6.8%	306833	34.78	7.5%	99.9*	30*	1.208	10351
3.70	387333	28359	28359	100.0%	9.7%	9.2%	387333	26.27	10.0%	99.8*	13*	0.982	13568
3.20	415298	33568	33571	100.0%	23.3%	22.8%	415298	10.94	24.3%	98.8*	5	0.857	16168
2.87	446197	38023	38029	100.0%	54.9%	54.9%	446197	4.43	57.4%	93.6*	1	0.787	18405
2.62	448622	41914	41927	100.0%	123.0%	126.3%	448621	1.81	129.1%	72.2*	1	0.721	20349
2.42	434785	45572	45748	99.6%	210.9%	221.1%	434771	0.91	222.9%	38.7*	0	0.662	22140
2.27	194443	34133	48991	69.7%	312.2%	330.9%	194364	0.40	344.8%	10.4*	2	0.618	15868
2.14	44307	15460	52251	29.6%	396.3%	428.0%	44194	0.19	494.7%	6.2	4	0.581	5408
total	2851812	271182	323058	83.9%	18.6%	18.6%	2851605	10.53	19.6%	99.5*	7	0.836	127756

Crystal 1 p2 720° 0.1s 0.2° χ 25°

SUBSET OF INTENSITY DATA WITH SIGNAL/NOISE >= -3.0 AS FUNCTION OF RESOLUTION

RESOLUTION LIMIT	NUMBER OF REFLECTIONS OBSERVED	UNIQUE	POSSIBLE	COMPLETENESS OF DATA	R-FACTOR observed	R-FACTOR expected	COMPARED	I/SIGMA	R-meas	CC(1/2)	Anomal Corr	SigAno	Nano
6.37	174323	12258	12286	99.8%	4.1%	4.0%	174323	55.63	4.3%	100.0*	61*	1.872	5516
4.52	307066	21983	21983	100.0%	7.3%	6.9%	307066	34.56	7.6%	99.9*	31*	1.207	10374
3.70	382617	28414	28414	100.0%	9.9%	9.4%	382617	25.65	10.3%	99.8*	13*	0.985	13595
3.20	418823	33635	33635	100.0%	24.5%	24.0%	418823	10.52	25.6%	98.8*	4	0.848	16203
2.87	453045	38073	38077	100.0%	58.0%	58.3%	453045	4.22	60.6%	93.0*	2	0.787	18428
2.62	448407	42035	42046	100.0%	131.9%	136.0%	448407	1.67	138.6%	69.1*	2	0.719	20407
2.42	432593	45734	45875	99.7%	230.9%	242.4%	432584	0.82	244.1%	35.5*	2	0.666	22235
2.27	189185	36160	49085	73.7%	331.5%	353.3%	189124	0.35	370.0%	8.8	2	0.607	16912
2.14	43962	16247	52361	31.0%	394.6%	423.0%	43844	0.18	500.8%	3.6	0	0.574	5468
total	2850021	274539	323762	84.8%	19.0%	19.0%	2849833	10.23	20.1%	99.5*	8	0.831	129138

Crystal 1 p2 720° 0.1s 0.2° χ 30°

SUBSET OF INTENSITY DATA WITH SIGNAL/NOISE >= -3.0 AS FUNCTION OF RESOLUTION

RESOLUTION LIMIT	NUMBER OF REFLECTIONS OBSERVED	UNIQUE	POSSIBLE	COMPLETENESS OF DATA	R-FACTOR observed	R-FACTOR expected	COMPARED	I/SIGMA	R-meas	CC(1/2)	Anomal Corr	SigAno	Nano
6.37	174056	12254	12283	99.8%	4.3%	4.1%	174056	53.88	4.5%	100.0*	60*	1.860	5515
4.52	306237	21959	21959	100.0%	7.6%	7.2%	306237	33.50	7.9%	99.9*	29*	1.197	10362
3.70	375892	28397	28397	100.0%	10.4%	9.8%	375892	24.52	10.8%	99.7*	12*	0.970	13587
3.20	414425	33613	33614	100.0%	25.9%	25.4%	414425	10.02	27.0%	98.6*	3	0.837	16191
2.87	470915	38040	38046	100.0%	61.8%	62.0%	470915	4.10	64.5%	92.7*	1	0.780	18414
2.62	461866	42002	42018	100.0%	141.1%	146.1%	461866	1.59	147.9%	67.5*	1	0.711	20389
2.42	427047	45707	45809	99.8%	241.8%	255.4%	427043	0.78	255.8%	33.1*	3	0.662	22225
2.27	184386	37859	49064	77.2%	345.4%	369.2%	184314	0.32	389.0%	8.9	3	0.602	17646
2.14	43516	16615	52347	31.7%	406.8%	439.3%	43418	0.17	521.7%	4.7	3	0.579	5517
total	2858340	276446	323537	85.4%	19.9%	19.9%	2858166	9.78	21.0%	99.4*	8	0.822	129846

Crystal 1 p2 720° 0.1s 0.2° χ 10° φ 90°

SUBSET OF INTENSITY DATA WITH SIGNAL/NOISE >= -3.0 AS FUNCTION OF RESOLUTION

RESOLUTION LIMIT	NUMBER OF REFLECTIONS OBSERVED	UNIQUE	POSSIBLE	COMPLETENESS OF DATA	R-FACTOR observed	R-FACTOR expected	COMPARED	I/SIGMA	R-meas	CC(1/2)	Anomal Corr	SigAno	Nano
6.37	173642	12258	12287	99.8%	4.2%	4.1%	173642	53.71	4.4%	100.0*	58*	1.813	5516
4.52	306360	21973	21973	100.0%	7.5%	7.1%	306360	33.34	7.8%	99.9*	29*	1.190	10371
3.70	385535	28405	28407	100.0%	10.4%	9.9%	385535	24.36	10.8%	99.7*	12*	0.970	13588
3.20	447401	33593	33594	100.0%	26.0%	25.4%	447401	10.25	27.0%	98.6*	3	0.840	16183
2.87	496423	37939	38085	99.6%	61.5%	61.2%	496422	4.10	64.0%	92.6*	0	0.778	18368
2.62	488345	40911	42024	97.4%	140.2%	143.2%	488339	1.61	146.4%	67.1*	0	0.707	19850
2.42	477677	42327	45818	92.4%	235.5%	246.0%	477673	0.84	246.7%	34.4*	-1	0.651	20561
2.27	238860	35241	49090	71.8%	389.5%	413.0%	238794	0.34	422.4%	9.4	1	0.599	16804
2.14	66765	14911	52304	28.5%	533.7%	569.0%	66693	0.17	608.4%	3.8	0	0.565	6277
total	3081008	267558	323582	82.7%	21.2%	21.2%	3080859	10.09	22.2%	99.5*	6	0.819	127518

-

Crystal 1 p2 8 × 720° 0.1s 0.2° XSCALE merged (χ 0°, χ 5°, χ 10°, χ 15°, χ 20°, χ 25°, χ 30°, χ 10°/φ 90°)

SUBSET OF INTENSITY DATA WITH SIGNAL/NOISE >= -3.0 AS FUNCTION OF RESOLUTION

RESOLUTION LIMIT	NUMBER OF REFLECTIONS OBSERVED	UNIQUE	POSSIBLE	COMPLETENESS OF DATA	R-FACTOR observed	R-FACTOR expected	COMPARED	I/SIGMA	R-meas	CC(1/2)	Anomal Corr	SigAno	Nano
6.37	1386659	12174	12202	99.8%	4.5%	4.7%	1386659	140.01	4.5%	100.0*	88*	4.325	5477
4.52	2454196	21888	21888	100.0%	7.7%	7.4%	2454196	93.15	7.7%	100.0*	71*	2.463	10329
3.70	3049586	28093	28094	100.0%	10.1%	9.8%	3049586	71.38	10.2%	100.0*	41*	1.560	13440
3.20	3383114	33947	33949	100.0%	24.4%	23.9%	3383114	30.83	24.5%	99.9*	17*	1.038	16349
2.87	3581359	37085	37089	100.0%	56.7%	56.7%	3581359	12.92	57.0%	99.2*	6	0.852	17943
2.62	3663331	41885	41906	99.9%	127.5%	129.3%	3663331	5.31	128.2%	95.7*	2	0.772	20325
2.42	3635948	47039	47049	100.0%	221.2%	228.3%	3635948	2.62	222.7%	83.2*	1	0.724	22882
2.27	1563282	44205	46995	94.1%	349.9%	365.7%	1563264	0.94	355.1%	25.8*	2	0.666	21434
2.14	417382	37444	52056	71.9%	477.3%	503.1%	417294	0.33	501.9%	4.6	1	0.606	17375
total	23134857	303760	321228	94.6%	20.7%	20.8%	23134751	25.26	20.9%	99.7*	15*	1.095	145554

—

Crystal 2 p2 720° 0.1s 0.2° χ 0°

SUBSET OF INTENSITY DATA WITH SIGNAL/NOISE >= -3.0 AS FUNCTION OF RESOLUTION

RESOLUTION LIMIT	NUMBER OF REFLECTIONS OBSERVED	UNIQUE	POSSIBLE	COMPLETENESS OF DATA	R-FACTOR observed	R-FACTOR expected	COMPARED	I/SIGMA	R-meas	CC(1/2)	Anomal Corr	SigAno	Nano
6.36	173903	12213	12242	99.8%	3.9%	3.9%	173903	57.13	4.0%	100.0*	64*	1.890	5496
4.51	307829	21907	21907	100.0%	7.0%	6.7%	307829	35.27	7.3%	99.9*	30*	1.183	10338
3.69	386038	28317	28318	100.0%	9.6%	9.3%	386038	25.84	10.0%	99.8*	12*	0.959	13550
3.20	443226	33525	33528	100.0%	24.9%	24.2%	443226	10.70	25.9%	98.8*	3	0.845	16147
2.86	488236	37759	37978	99.4%	60.1%	59.5%	488233	4.28	62.5%	93.4*	1	0.779	18228
2.61	481702	40426	41898	96.5%	138.7%	140.4%	481700	1.72	144.8%	70.8*	0	0.708	19476
2.42	481778	41866	45685	91.6%	230.8%	238.2%	481768	0.92	241.5%	39.6*	2	0.665	20183
2.26	237279	38371	48895	78.5%	385.9%	405.3%	237231	0.35	422.5%	9.7	1	0.597	18365
2.14	66862	17342	52214	33.2%	473.4%	499.1%	66743	0.19	552.4%	4.4	2	0.584	5852
total	3066853	271726	322665	84.2%	20.1%	20.1%	3066671	10.48	21.1%	99.6*	7	0.823	127635

Crystal 2 p2 720° 0.1s 0.2° χ 10°

SUBSET OF INTENSITY DATA WITH SIGNAL/NOISE >= -3.0 AS FUNCTION OF RESOLUTION

RESOLUTION LIMIT	NUMBER OF REFLECTIONS OBSERVED	UNIQUE	POSSIBLE	COMPLETENESS OF DATA	R-FACTOR observed	R-FACTOR expected	COMPARED	I/SIGMA	R-meas	CC(1/2)	Anomal Corr	SigAno	Nano
6.36	173158	12209	12238	99.8%	4.0%	4.0%	173158	55.69	4.1%	100.0*	60*	1.826	5494
4.51	305873	21909	21909	100.0%	7.1%	6.8%	305873	34.65	7.3%	99.9*	28*	1.163	10339
3.69	384157	28317	28318	100.0%	9.7%	9.4%	384157	25.47	10.1%	99.8*	12*	0.950	13549
3.20	440774	33513	33516	100.0%	25.1%	24.5%	440774	10.55	26.1%	98.8*	5	0.847	16139
2.86	486566	37733	37978	99.4%	61.0%	60.3%	486565	4.20	63.5%	93.1*	1	0.778	18211
2.61	482104	40551	41900	96.8%	141.8%	143.3%	482091	1.66	148.1%	69.1*	0	0.704	19516
2.42	482535	42149	45679	92.3%	233.9%	241.7%	482506	0.90	244.9%	37.0*	1	0.653	20265
2.26	235035	39377	48877	80.6%	389.3%	409.2%	234980	0.34	427.8%	8.6	1	0.602	18879
2.13	66727	17803	52218	34.1%	484.6%	515.8%	66488	0.18	567.9%	4.0	2	0.580	5842
total	3056929	273561	322633	84.8%	20.2%	20.2%	3056592	10.21	21.2%	99.6*	7	0.815	128234

Crystal 2 p2 720° 0.1s 0.2° χ 20°

SUBSET OF INTENSITY DATA WITH SIGNAL/NOISE >= -3.0 AS FUNCTION OF RESOLUTION

RESOLUTION LIMIT	NUMBER OF REFLECTIONS OBSERVED	UNIQUE	POSSIBLE	COMPLETENESS OF DATA	R-FACTOR observed	R-FACTOR expected	COMPARED	I/SIGMA	R-meas	CC(1/2)	Anomal Corr	SigAno	Nano
6.35	172774	12202	12231	99.8%	4.2%	4.1%	172774	53.87	4.3%	100.0*	59*	1.818	5491
4.51	304631	21878	21878	100.0%	7.3%	6.9%	304631	33.97	7.5%	99.9*	29*	1.181	10324
3.69	383938	28325	28326	100.0%	9.7%	9.4%	383938	25.44	10.1%	99.8*	12*	0.969	13553
3.19	442691	33484	33488	100.0%	24.1%	23.6%	442691	10.90	25.1%	98.8*	4	0.852	16126
2.86	485814	37913	37947	99.9%	57.1%	56.9%	485812	4.40	59.4%	93.7*	1	0.779	18339
2.61	481488	41081	41889	98.1%	129.8%	132.1%	481477	1.77	135.7%	71.0*	0	0.710	19873
2.42	482840	42685	45647	93.5%	215.7%	223.7%	482800	0.96	225.9%	40.4*	0	0.660	20575
2.26	239181	36284	48830	74.3%	357.5%	377.4%	238991	0.40	388.7%	12.0*	1	0.606	17098
2.13	67380	16719	52171	32.0%	467.8%	499.0%	67098	0.21	541.0%	4.7	1	0.582	5883
total	3060737	270571	322407	83.9%	19.9%	19.9%	3060212	10.29	20.8%	99.6*	7	0.824	127262

Crystal 2 p2 720° 0.1s 0.2° χ 30°

SUBSET OF INTENSITY DATA WITH SIGNAL/NOISE >= -3.0 AS FUNCTION OF RESOLUTION

RESOLUTION LIMIT	NUMBER OF REFLECTIONS OBSERVED	UNIQUE	POSSIBLE	COMPLETENESS OF DATA	R-FACTOR observed	R-FACTOR expected	COMPARED	I/SIGMA	R-meas	CC(1/2)	Anomal Corr	SigAno	Nano
6.35	173520	12221	12249	99.8%	4.2%	4.1%	173520	53.66	4.3%	100.0*	60*	1.804	5499
4.51	304481	21908	21908	100.0%	7.4%	7.0%	304481	33.58	7.6%	99.9*	31*	1.184	10339
3.68	386467	28329	28332	100.0%	9.8%	9.5%	386467	25.13	10.2%	99.8*	13*	0.960	13555
3.19	443458	33547	33551	100.0%	25.0%	24.3%	443458	10.47	26.0%	98.7*	11*	0.871	16156
2.86	486263	37998	38001	100.0%	60.0%	59.9%	486263	4.15	62.5%	93.1*	3	0.802	18388
2.61	479471	41772	41902	99.7%	138.4%	141.2%	479468	1.61	144.9%	67.7*	1	0.704	20283
2.42	484720	44390	45719	97.1%	230.2%	240.1%	484679	0.86	241.6%	34.1*	0	0.647	21551
2.26	234949	30855	48911	63.1%	369.8%	391.2%	234735	0.40	396.9%	12.8*	1	0.606	14543
2.13	66160	14512	52227	27.8%	449.9%	481.3%	65918	0.23	510.0%	3.6	2	0.589	6318
total	3059489	265532	322800	82.3%	20.0%	20.0%	3058989	10.29	21.0%	99.6*	8	0.827	126632

Crystal 2 p2 720° 0.1s 0.2° χ 25°

SUBSET OF INTENSITY DATA WITH SIGNAL/NOISE >= -3.0 AS FUNCTION OF RESOLUTION

RESOLUTION LIMIT	NUMBER OF REFLECTIONS OBSERVED	UNIQUE	POSSIBLE	COMPLETENESS OF DATA	R-FACTOR observed	R-FACTOR expected	COMPARED	I/SIGMA	R-meas	CC(1/2)	Anomal Corr	SigAno	Nano
6.35	173180	12204	12233	99.8%	4.1%	4.1%	173180	54.12	4.3%	100.0*	60*	1.826	5492
4.51	303745	21891	21891	100.0%	7.3%	6.9%	303745	33.70	7.6%	99.9*	29*	1.179	10330
3.69	384113	28323	28323	100.0%	9.8%	9.5%	384113	25.07	10.1%	99.8*	14*	0.959	13552
3.20	444347	33512	33513	100.0%	25.1%	24.5%	444347	10.47	26.1%	98.7*	11*	0.864	16140
2.86	485805	37954	37957	100.0%	60.7%	60.4%	485805	4.12	63.2%	93.0*	2	0.784	18368
2.61	483027	41508	41889	99.1%	140.7%	143.2%	483023	1.61	147.2%	67.0*	0	0.699	20133
2.42	486154	43434	45663	95.1%	235.0%	244.0%	486144	0.86	246.4%	35.3*	2	0.653	21055
2.26	241478	33831	48864	69.2%	378.6%	399.4%	241405	0.38	408.7%	11.4*	2	0.611	16010
2.13	67572	15372	52185	29.5%	511.2%	542.7%	67470	0.19	584.1%	2.2	1	0.576	6265
total	3069421	268029	322518	83.1%	20.1%	20.2%	3069232	10.20	21.1%	99.6*	8	0.823	127345

Crystal 2 p2 720° 0.1s 0.2° χ 15°

SUBSET OF INTENSITY DATA WITH SIGNAL/NOISE >= -3.0 AS FUNCTION OF RESOLUTION

RESOLUTION LIMIT	NUMBER OF REFLECTIONS OBSERVED	UNIQUE	POSSIBLE	COMPLETENESS OF DATA	R-FACTOR observed	R-FACTOR expected	COMPARED	I/SIGMA	R-meas	CC(1/2)	Anomal Corr	SigAno	Nano
6.36	172878	12212	12241	99.8%	4.1%	4.0%	172878	54.92	4.2%	100.0*	60*	1.815	5496
4.51	304659	21887	21887	100.0%	7.2%	6.8%	304659	34.12	7.5%	99.9*	30*	1.183	10328
3.69	384473	28328	28328	100.0%	9.8%	9.5%	384473	25.20	10.2%	99.8*	13*	0.962	13554
3.20	441755	33512	33516	100.0%	25.1%	24.5%	441755	10.43	26.1%	98.8*	5	0.857	16139
2.86	488113	37854	37966	99.7%	61.1%	60.9%	488113	4.10	63.6%	93.1*	2	0.777	18298
2.61	483044	40793	41884	97.4%	142.9%	145.1%	483041	1.62	149.3%	67.6*	0	0.702	19668
2.42	484430	42245	45678	92.5%	240.2%	249.2%	484424	0.85	251.5%	34.6*	1	0.652	20354
2.26	240892	38885	48871	79.6%	401.4%	422.3%	240840	0.33	439.2%	9.2	1	0.599	18636
2.13	68338	17868	52198	34.2%	512.9%	546.4%	68232	0.17	599.7%	5.3	3	0.577	6016
total	3068582	273584	322569	84.8%	20.1%	20.1%	3068415	10.07	21.0%	99.6*	7	0.817	128489

Crystal 2 p2 720° 0.1s 0.2° χ 5°

SUBSET OF INTENSITY DATA WITH SIGNAL/NOISE >= -3.0 AS FUNCTION OF RESOLUTION

RESOLUTION LIMIT	NUMBER OF REFLECTIONS OBSERVED	UNIQUE	POSSIBLE	COMPLETENESS OF DATA	R-FACTOR observed	R-FACTOR expected	COMPARED	I/SIGMA	R-meas	CC(1/2)	Anomal Corr	SigAno	Nano
6.36	173185	12210	12239	99.8%	4.0%	3.9%	173185	55.90	4.1%	100.0*	60*	1.815	5495
4.52	305797	21883	21883	100.0%	7.2%	6.8%	305797	34.34	7.4%	99.9*	28*	1.159	10326
3.69	385883	28331	28331	100.0%	9.9%	9.5%	385883	25.06	10.2%	99.8*	9	0.930	13555
3.20	444478	33517	33518	100.0%	25.8%	25.1%	444478	10.23	26.8%	98.7*	4	0.845	16142
2.86	491967	37749	37952	99.5%	63.3%	63.0%	491965	4.01	65.8%	92.8*	2	0.779	18218
2.61	484966	40425	41890	96.5%	150.1%	152.6%	484962	1.55	156.8%	66.7*	0	0.698	19480
2.42	486144	41948	45679	91.8%	253.1%	262.1%	486141	0.82	264.9%	32.5*	2	0.651	20226
2.26	244048	39620	48858	81.1%	422.5%	445.1%	244011	0.30	462.8%	8.0	2	0.596	19011
2.14	69148	18152	52185	34.8%	517.5%	547.2%	69026	0.16	605.4%	40.0*	1	0.573	6082
total	3085616	273835	322535	84.9%	20.3%	20.3%	3085448	10.04	21.3%	99.6*	7	0.809	128535

Crystal 2 p2 720° 0.1s 0.2° χ 15° φ 90°

SUBSET OF INTENSITY DATA WITH SIGNAL/NOISE >= -3.0 AS FUNCTION OF RESOLUTION

RESOLUTION LIMIT	NUMBER OF REFLECTIONS OBSERVED	UNIQUE	POSSIBLE	COMPLETENESS OF DATA	R-FACTOR observed	R-FACTOR expected	COMPARED	I/SIGMA	R-meas	CC(1/2)	Anomal Corr	SigAno	Nano
6.35	173149	12241	12270	99.8%	4.1%	4.1%	173149	54.37	4.2%	100.0*	59*	1.802	5508
4.51	306292	21993	21993	100.0%	7.3%	7.0%	306292	33.50	7.6%	99.9*	27*	1.171	10380
3.69	384740	28377	28378	100.0%	10.4%	10.1%	384740	24.06	10.8%	99.8*	11*	0.954	13580
3.20	447051	33649	33651	100.0%	28.1%	27.4%	447051	9.63	29.3%	98.5*	4	0.849	16204
2.86	493543	38076	38083	100.0%	71.4%	71.0%	493543	3.61	74.3%	91.2*	2	0.778	18431
2.61	484154	42007	42024	100.0%	173.9%	176.8%	484154	1.32	181.9%	59.5*	0	0.691	20390
2.42	478964	45468	45840	99.2%	306.2%	317.1%	478961	0.63	322.0%	25.8*	1	0.634	22091
2.26	240310	40114	49099	81.7%	454.0%	476.8%	240271	0.27	498.2%	7.1	2	0.586	18586
2.13	67136	17683	52299	33.8%	556.0%	590.2%	67017	0.16	650.2%	4.8	4	0.583	5451
total	3075339	279608	323637	86.4%	21.5%	21.5%	3075178	9.46	22.6%	99.5*	7	0.806	130621

-

Crystal 2 p2 8 × 720° 0.1s 0.2° XSCALE merged (χ 0°, χ 10°, χ 20°, χ 30°, χ 25°, χ 15°, χ 5°, χ 15°/φ 90°)

SUBSET OF INTENSITY DATA WITH SIGNAL/NOISE >= -3.0 AS FUNCTION OF RESOLUTION

RESOLUTION LIMIT	NUMBER OF REFLECTIONS OBSERVED	UNIQUE	POSSIBLE	COMPLETENESS OF DATA	R-FACTOR observed	R-FACTOR expected	COMPARED	I/SIGMA	R-meas	CC(1/2)	Anomal Corr	SigAno	Nano
6.35	1390358	12264	12292	99.8%	4.3%	4.7%	1390358	140.27	4.4%	100.0*	89*	4.216	5518
4.51	2451263	21970	21970	100.0%	7.6%	7.4%	2451263	92.23	7.6%	100.0*	68*	2.307	10370
3.69	3070190	28253	28255	100.0%	10.4%	10.1%	3070190	68.99	10.4%	100.0*	37*	1.469	13518
3.20	3531429	33383	33384	100.0%	26.2%	25.6%	3531429	29.61	26.3%	99.8*	13*	1.002	16078
2.86	3949978	38418	38423	100.0%	64.0%	63.7%	3949978	11.75	64.3%	99.1*	6	0.841	18591
2.61	3892128	42284	42303	100.0%	151.3%	152.9%	3892128	4.52	152.1%	93.9*	1	0.752	20522
2.42	3804205	44743	45026	99.4%	254.5%	261.2%	3804202	2.30	256.1%	71.0*	1	0.700	21742
2.26	1948183	49418	50419	98.0%	430.2%	447.5%	1948177	0.81	436.0%	23.7*	1	0.647	23979
2.13	503924	39610	52937	74.8%	563.3%	590.6%	503703	0.30	588.4%	4.1	3	0.613	16926
total	24541658	310343	325009	95.5%	21.9%	22.0%	24541428	24.11	22.1%	99.8*	13*	1.055	147244

—

Crystal 3 p2 720° 0.1s 0.2° χ 0°

SUBSET OF INTENSITY DATA WITH SIGNAL/NOISE >= -3.0 AS FUNCTION OF RESOLUTION

RESOLUTION LIMIT	NUMBER OF REFLECTIONS OBSERVED	UNIQUE	POSSIBLE	COMPLETENESS OF DATA	R-FACTOR observed	R-FACTOR expected	COMPARED	I/SIGMA	R-meas	CC(1/2)	Anomal Corr	SigAno	Nano
6.29	181082	12674	12707	99.7%	4.3%	4.2%	181082	52.76	4.4%	100.0*	67*	1.778	5711
4.46	318153	22779	22780	100.0%	8.5%	8.3%	318153	29.20	8.8%	99.8*	23*	1.078	10761
3.65	402136	29379	29380	100.0%	15.5%	15.0%	402136	17.52	16.1%	99.6*	9	0.903	14064
3.16	464999	34734	34745	100.0%	48.1%	46.1%	464997	5.77	50.0%	96.4*	3	0.834	16739
2.83	500557	39032	39407	99.0%	137.8%	136.1%	500547	1.81	143.5%	74.9*	1	0.731	18878
2.58	511965	41725	43645	95.6%	341.9%	347.9%	511955	0.63	356.7%	28.0*	1	0.636	20185
2.39	432300	43286	47383	91.4%	602.9%	620.8%	432286	0.27	635.8%	6.7	1	0.586	20949
2.24	234791	39767	50839	78.2%	838.0%	869.1%	234689	0.12	922.4%	3.5	1	0.556	18828
2.11	50308	16829	54127	31.1%	739.3%	776.1%	50124	0.10	911.9%	3.8	4	0.570	6182
total	3096291	280205	335013	83.6%	30.0%	29.8%	3095969	7.72	31.5%	99.2*	7	0.766	132297

Crystal 3 p2 720° 0.1s 0.2° χ 10°

SUBSET OF INTENSITY DATA WITH SIGNAL/NOISE >= -3.0 AS FUNCTION OF RESOLUTION

RESOLUTION LIMIT	NUMBER OF REFLECTIONS OBSERVED	UNIQUE	POSSIBLE	COMPLETENESS OF DATA	R-FACTOR observed	R-FACTOR expected	COMPARED	I/SIGMA	R-meas	CC(1/2)	Anomal Corr	SigAno	Nano
6.29	181263	12675	12704	99.8%	4.3%	4.3%	181263	52.20	4.5%	100.0*	60*	1.777	5710
4.46	318111	22747	22748	100.0%	8.6%	8.4%	318111	28.82	9.0%	99.8*	20*	1.064	10745
3.65	402120	29370	29371	100.0%	15.7%	15.3%	402120	17.13	16.4%	99.5*	8	0.890	14060
3.16	463760	34711	34720	100.0%	49.6%	47.8%	463760	5.53	51.6%	96.0*	2	0.824	16727
2.83	500209	39381	39401	99.9%	143.1%	142.0%	500208	1.71	149.1%	71.9*	1	0.725	19070
2.58	514632	42978	43583	98.6%	357.2%	363.1%	514626	0.59	373.1%	24.7*	1	0.636	20865
2.39	434771	45044	47384	95.1%	636.7%	654.7%	434756	0.24	673.0%	6.8	1	0.579	21847
2.24	234884	37588	50791	74.0%	870.7%	904.3%	234783	0.12	952.0%	3.2	2	0.556	18030
2.11	51425	15684	54085	29.0%	701.6%	730.3%	51283	0.11	845.2%	2.5	1	0.557	6895
total	3101175	280178	334787	83.7%	30.4%	30.3%	3100910	7.58	32.0%	99.2*	6	0.758	133949

Crystal 3 p2 720° 0.1s 0.2°χ 20°

SUBSET OF INTENSITY DATA WITH SIGNAL/NOISE >= -3.0 AS FUNCTION OF RESOLUTION

RESOLUTION LIMIT	NUMBER OF REFLECTIONS OBSERVED	UNIQUE	POSSIBLE	COMPLETENESS OF DATA	R-FACTOR observed	R-FACTOR expected	COMPARED	I/SIGMA	R-meas	CC(1/2)	Anomal Corr	SigAno	Nano
6.29	180797	12665	12697	99.7%	4.6%	4.5%	180796	50.29	4.8%	100.0*	55*	1.710	5704
4.46	316914	22729	22730	100.0%	9.2%	8.9%	316914	27.71	9.6%	99.8*	22*	1.066	10735
3.65	400743	29348	29348	100.0%	16.7%	16.1%	400743	16.54	17.3%	99.5*	7	0.897	14049
3.16	460266	34689	34694	100.0%	51.3%	49.6%	460266	5.32	53.4%	95.8*	3	0.824	16714
2.83	496968	39345	39361	100.0%	147.6%	147.1%	496968	1.64	153.8%	70.9*	2	0.722	19049
2.58	514622	43530	43553	99.9%	366.5%	374.9%	514622	0.56	383.0%	24.5*	1	0.629	21135
2.39	435216	46932	47342	99.1%	613.9%	634.2%	435210	0.25	650.3%	6.9	2	0.584	22780
2.24	236236	39568	50735	78.0%	806.1%	838.5%	236118	0.13	885.9%	3.5	1	0.562	18457
2.11	51494	17184	54036	31.8%	730.6%	761.3%	51271	0.10	900.6%	2.7	1	0.558	5686
total	3093256	285990	334496	85.5%	31.8%	31.8%	3092908	7.15	33.5%	99.0*	6	0.755	134309

Crystal 3 p2 720° 0.1s 0.2° χ 30°

SUBSET OF INTENSITY DATA WITH SIGNAL/NOISE >= -3.0 AS FUNCTION OF RESOLUTION

RESOLUTION LIMIT	NUMBER OF REFLECTIONS OBSERVED	UNIQUE	POSSIBLE	COMPLETENESS OF DATA	R-FACTOR observed	R-FACTOR expected	COMPARED	I/SIGMA	R-meas	CC(1/2)	Anomal Corr	SigAno	Nano
6.29	180137	12627	12656	99.8%	4.3%	4.2%	180137	52.67	4.5%	100.0*	60*	1.813	5686
4.47	314620	22661	22661	100.0%	8.5%	8.2%	314620	29.24	8.8%	99.8*	22*	1.086	10704
3.65	399778	29252	29252	100.0%	14.8%	14.3%	399778	17.97	15.4%	99.6*	8	0.902	14000
3.17	462534	34556	34565	100.0%	45.1%	44.0%	462534	6.04	46.9%	96.7*	2	0.821	16653
2.83	488239	39276	39296	99.9%	128.8%	128.6%	488239	1.91	134.3%	75.1*	2	0.735	19017
2.59	505821	43417	43436	100.0%	316.2%	322.9%	505821	0.67	330.7%	29.5*	1	0.645	21071
2.40	427153	47145	47184	99.9%	545.6%	562.0%	427153	0.29	578.8%	7.5	2	0.591	22944
2.24	238392	42127	50545	83.3%	721.0%	750.2%	238327	0.15	796.8%	3.6	2	0.571	19951
2.11	53011	18519	53947	34.3%	637.7%	671.0%	52839	0.11	794.9%	4.5	4	0.568	6309
total	3069685	289580	333542	86.8%	28.7%	28.7%	3069448	7.56	30.2%	99.2*	6	0.765	136335

Crystal 3 p2 720° 0.1s 0.2° χ 25°

SUBSET OF INTENSITY DATA WITH SIGNAL/NOISE >= -3.0 AS FUNCTION OF RESOLUTION

RESOLUTION LIMIT	NUMBER OF REFLECTIONS OBSERVED	UNIQUE	POSSIBLE	COMPLETENESS OF DATA	R-FACTOR observed	R-FACTOR expected	COMPARED	I/SIGMA	R-meas	CC(1/2)	Anomal Corr	SigAno	Nano
6.29	180770	12661	12693	99.7%	4.4%	4.3%	180770	52.01	4.5%	100.0*	56*	1.744	5702
4.47	315458	22721	22721	100.0%	8.7%	8.4%	315458	28.66	9.0%	99.8*	24*	1.085	10732
3.65	400557	29335	29337	100.0%	15.5%	15.0%	400557	17.27	16.1%	99.6*	8	0.902	14043
3.16	460062	34673	34682	100.0%	48.1%	46.8%	460062	5.63	50.0%	96.1*	4	0.826	16708
2.83	492523	39312	39334	99.9%	139.9%	139.7%	492523	1.75	145.9%	72.4*	2	0.723	19028
2.59	512789	43511	43545	99.9%	349.8%	356.6%	512789	0.60	365.7%	26.6*	0	0.627	21125
2.39	431679	47234	47316	99.8%	593.6%	611.4%	431677	0.26	629.6%	5.9	2	0.588	22967
2.24	235456	41077	50697	81.0%	768.2%	797.4%	235383	0.14	847.9%	7.3	3	0.564	19358
2.11	51835	17729	54052	32.8%	747.3%	778.5%	51675	0.09	926.8%	2.7	4	0.565	5948
total	3081129	288253	334377	86.2%	29.8%	29.8%	3080894	7.37	31.4%	99.1*	6	0.758	135611

Crystal 3 p2 720° 0.1s 0.2° χ 15°

SUBSET OF INTENSITY DATA WITH SIGNAL/NOISE >= -3.0 AS FUNCTION OF RESOLUTION

RESOLUTION LIMIT	NUMBER OF REFLECTIONS OBSERVED	UNIQUE	POSSIBLE	COMPLETENESS OF DATA	R-FACTOR observed	R-FACTOR expected	COMPARED	I/SIGMA	R-meas	CC(1/2)	Anomal Corr	SigAno	Nano
6.29	181308	12684	12715	99.8%	4.4%	4.3%	181308	51.55	4.5%	100.0*	58*	1.734	5713
4.46	318956	22822	22822	100.0%	8.8%	8.6%	318956	28.23	9.2%	99.8*	22*	1.054	10783
3.65	401975	29383	29386	100.0%	16.1%	15.6%	401975	16.67	16.7%	99.5*	9	0.905	14063
3.16	463165	34808	34814	100.0%	51.5%	49.9%	463165	5.28	53.5%	95.7*	3	0.819	16774
2.83	498477	39404	39421	100.0%	152.3%	151.4%	498477	1.61	158.7%	69.1*	2	0.726	19076
2.58	517319	43598	43695	99.8%	386.1%	392.7%	517318	0.53	403.5%	22.0*	1	0.629	21165
2.39	433348	46198	47409	97.4%	655.1%	672.7%	433342	0.23	693.8%	4.5	1	0.576	22387
2.24	235320	38240	50876	75.2%	826.5%	851.9%	235228	0.13	905.1%	3.0	1	0.555	17832
2.11	50852	16536	54210	30.5%	753.6%	787.1%	50716	0.09	922.2%	2.7	1	0.551	5979
total	3100720	283673	335348	84.6%	31.0%	30.9%	3100485	7.31	32.6%	99.1*	6	0.755	133772

Crystal 3 p2 720° 0.1s 0.2° χ 5°

SUBSET OF INTENSITY DATA WITH SIGNAL/NOISE >= -3.0 AS FUNCTION OF RESOLUTION

RESOLUTION LIMIT	NUMBER OF REFLECTIONS OBSERVED	UNIQUE	POSSIBLE	COMPLETENESS OF DATA	R-FACTOR observed	R-FACTOR expected	COMPARED	I/SIGMA	R-meas	CC(1/2)	Anomal Corr	SigAno	Nano
6.29	181913	12714	12744	99.8%	4.7%	4.7%	181913	47.80	4.9%	100.0*	53*	1.627	5728
4.46	320474	22861	22861	100.0%	9.9%	9.7%	320474	25.52	10.3%	99.8*	19*	1.023	10802
3.65	404095	29462	29464	100.0%	19.0%	18.4%	404095	14.59	19.7%	99.4*	4	0.872	14100
3.16	467058	34858	34863	100.0%	63.3%	60.7%	467058	4.40	65.8%	93.7*	2	0.804	16799
2.83	501180	39407	39531	99.7%	187.7%	186.4%	501176	1.28	195.5%	58.5*	1	0.699	19079
2.58	512608	42492	43765	97.1%	482.7%	491.8%	512559	0.40	503.9%	14.7*	0	0.604	20575
2.39	431534	44146	47482	93.0%	816.9%	841.7%	431501	0.16	862.5%	3.3	1	0.560	21371
2.24	237656	39101	51010	76.7%	1021.1%	1055.2%	237490	0.08	1119.5%	1.5	1	0.544	18707
2.11	50448	15870	54350	29.2%	842.6%	883.0%	50136	0.08	1020.8%	2.0	3	0.551	6630
total	3106966	280911	336070	83.6%	36.2%	36.1%	3106402	6.60	38.0%	98.9*	5	0.731	133791

Crystal 3 p2 720° 0.1s 0.2° χ 10° φ 90°

SUBSET OF INTENSITY DATA WITH SIGNAL/NOISE >= -3.0 AS FUNCTION OF RESOLUTION

RESOLUTION LIMIT	NUMBER OF REFLECTIONS OBSERVED	UNIQUE	POSSIBLE	COMPLETENESS OF DATA	R-FACTOR observed	R-FACTOR expected	COMPARED	I/SIGMA	R-meas	CC(1/2)	Anomal Corr	SigAno	Nano
6.29	180898	12686	12719	99.7%	4.6%	4.5%	180898	49.30	4.8%	99.9*	54*	1.636	5714
4.46	318324	22821	22824	100.0%	9.3%	9.0%	318324	26.75	9.7%	99.8*	18*	1.027	10784
3.65	396153	29320	29405	99.7%	17.4%	16.8%	396153	15.30	18.1%	99.4*	5	0.879	14030
3.16	444124	34035	34839	97.7%	57.3%	55.6%	444116	4.63	59.6%	94.5*	3	0.813	16332
2.83	479964	37633	39452	95.4%	176.7%	176.1%	479960	1.38	184.0%	62.8*	1	0.700	18104
2.58	498867	39910	43716	91.3%	447.7%	455.4%	498859	0.46	466.7%	18.0*	0	0.610	19220
2.39	423654	40741	47417	85.9%	752.8%	774.5%	423641	0.19	792.2%	6.0	3	0.574	19604
2.24	226166	35493	50912	69.7%	953.9%	986.2%	226092	0.09	1041.3%	3.0	3	0.551	17001
2.11	51587	16123	54257	29.7%	856.5%	888.6%	51451	0.08	1039.9%	2.3	-1	0.549	6268
total	3019737	268762	335541	80.1%	32.6%	32.5%	3019494	7.16	34.2%	99.2*	5	0.744	127057

_

Crystal 3 p2 8 × 720° 0.1s 0.2° XSCALE merged (χ 0°, χ 10°, χ 20°, χ 30°, χ 25°, χ 15°, χ 5°, χ 10°/φ 90°)

SUBSET OF INTENSITY DATA WITH SIGNAL/NOISE >= -3.0 AS FUNCTION OF RESOLUTION

RESOLUTION LIMIT	NUMBER OF REFLECTIONS OBSERVED	UNIQUE	POSSIBLE	COMPLETENESS OF DATA	R-FACTOR observed	R-FACTOR expected	COMPARED	I/SIGMA	R-meas	CC(1/2)	Anomal Corr	SigAno	Nano
6.29	1446090	12657	12695	99.7%	4.8%	5.1%	1446090	131.10	4.9%	100.0*	86*	3.873	5700
4.46	2554322	22885	22885	100.0%	9.4%	9.3%	2554322	76.32	9.5%	100.0*	56*	1.912	10812
3.65	3195911	29264	29264	100.0%	17.0%	16.6%	3195911	46.24	17.1%	99.9*	24*	1.168	14007
3.16	3718340	35039	35050	100.0%	53.5%	51.8%	3718340	15.13	53.7%	99.5*	7	0.883	16880
2.83	3929031	39105	39125	99.9%	157.2%	155.5%	3929031	4.66	157.9%	95.2*	3	0.779	18924
2.58	4173628	44511	44542	99.9%	396.4%	400.4%	4173628	1.55	398.5%	70.8*	2	0.682	21600
2.39	3409745	47237	47268	99.9%	694.1%	710.5%	3409745	0.62	699.0%	25.5*	1	0.624	22978
2.24	1821353	49067	49542	99.0%	934.7%	962.0%	1821342	0.27	947.9%	3.8	1	0.587	23899
2.11	417365	41814	55074	75.9%	935.4%	971.3%	417107	0.13	989.2%	1.6	1	0.566	18657
total	24665785	321579	335445	95.9%	34.4%	34.3%	24665516	17.38	34.6%	99.4*	10	0.928	153457
